# Armadillo repeat containing 12 promotes neuroblastoma progression through interaction with retinoblastoma binding protein 4

**DOI:** 10.1038/s41467-018-05286-2

**Published:** 2018-07-19

**Authors:** Dan Li, Huajie Song, Hong Mei, Erhu Fang, Xiaojing Wang, Feng Yang, Huanhuan Li, Yajun Chen, Kai Huang, Liduan Zheng, Qiangsong Tong

**Affiliations:** 1 Department of Pediatric Surgery, Union Hospital, Tongji Medical College, Huazhong University of Science and Technology, 1277 Jiefang Avenue, 430022 Wuhan, Hubei Province P.R. China; 2 Department of Pathology, Union Hospital, Tongji Medical College, Huazhong University of Science and Technology, 1277 Jiefang Avenue, 430022 Wuhan, Hubei Province P.R. China; 3 Clinical Center of Human Genomic Research, Union Hospital, Tongji Medical College, Huazhong University of Science and Technology, 1277 Jiefang Avenue, 430022 Wuhan, Hubei Province P.R. China

## Abstract

Recent studies suggest the emerging roles of armadillo (ARM) family proteins in tumor progression. However, the functions and underlying mechanisms of ARM members in tumorigenesis and aggressiveness of neuroblastoma (NB) remain to be determined. Herein, we identify armadillo repeat containing 12 (*ARMC12*) as an ARM member associated with NB progression. *ARMC12* promotes the growth and aggressiveness of NB cell lines. Mechanistically, ARMC12 physically interacts with retinoblastoma binding protein 4 (RBBP4) to facilitate the formation and activity of polycomb repressive complex 2, resulting in transcriptional repression of tumor suppressive genes. Blocking the interaction between ARMC12 and RBBP4 by cell-penetrating inhibitory peptide activates the downstream gene expression and suppresses the tumorigenesis and aggressiveness of NB cells. Both *ARMC12* and *RBBP4* are upregulated in NB tissues, and are associated with unfavorable outcome of patients. These findings suggest the crucial roles of *ARMC12* in tumor progression and a potential therapeutic approach for NB.

## Abstract (web summary)

Armadillo (ARM) family proteins can act as oncogenes or tumor suppressors. Here, the authors show that a new ARM protein (ARMC12) is upregulated in neuroblastoma, binds the PRC2 component RBBP4, and inhibits transcription of tumor suppressive genes.

## Introduction

Neuroblastoma (NB) is one of the most common malignant solid tumors in pediatric population that arises from neural crest-derived cells, and constitutes 15% of cancer-related mortality in childhood. The clinical courses of NB are remarkably heterogeneous, including spontaneous remission or rapid progression and death^1^. For patients suffering from high-risk NB, the clinical outcome remains poor despite the application of multimodal therapies^2^. Although numerous genetic abnormalities, such as gain of chromosome regions 1q and 17q, *MYCN* amplification, and loss of heterozygosity at chromosome regions 1p, 3p, and 11q, have been proposed as indicators of poor prognosis^3^, the mechanisms essential for the aggressiveness and progression of NB still warrant further investigation to ameliorate the outcome of patients.

Previous studies have shown that armadillo (ARM) family proteins, featured by the existence of 42 amino acid motif repeats, play crucial roles in tumorigenesis and aggressiveness. For example, *β-catenin*^4^, *δ-catenin*^5,6^, and *p120*^7^ possess oncogenic activities, yet adenoma polyposis coli (*APC*) and armadillo repeat-containing X-linked protein 1 (*ARMCX1*) act as tumor suppressors^8^. As an ARM family member, β-catenin exerts important functions in regulating Wnt signaling and adherence junctions through interaction with lymphoid enhancer factor/T-cell factor and E-cadherin^9^, while APC regulates the Wnt signaling via synergistically acting with glycogen synthase kinase 3β, casein kinase Ι, and axin in degrading β-catenin^10,11^. Overexpression of *ARMCX1* suppresses the anchorage-dependent and -independent growth of colon cancer cells^12^, while knockdown of *ARMCX1* accelerates the hepatocarcinogenesis in mice^13^, indicating the tumor suppressive roles of *ARMCX1*. Thus, it has been an urgent task to determine the functions of ARM repeat-containing proteins in the aggressiveness and progression of tumors.

In the current study, we identify armadillo repeat-containing 12 (*ARMC12*) as an ARM family member associated with unfavorable outcome of NB patients through comprehensive analysis of public datasets. We demonstrate that *ARMC12* is highly expressed in clinical NB specimens, and drives the growth and aggressiveness of NB cell lines. Mechanistical studies show that ARMC12 physically interacts with retinoblastoma binding protein 4 (RBBP4) to facilitate the formation of polycomb repressive complex 2 (PRC2) and increase the histone methyltransferase activity of enhancer of zeste 2 polycomb repressive complex 2 subunit (EZH2), resulting in transcriptional repression of tumor suppressive genes. In addition, cell-penetrating inhibitory peptide blocking the interaction between ARMC12 and RBBP4 suppresses the tumorigenesis and aggressiveness of NB cells, suggesting the essential functions of *ARMC12* in NB progression.

## Results

### *ARMC12* is associated with the progression and outcome of NB

**Fig. 1:**
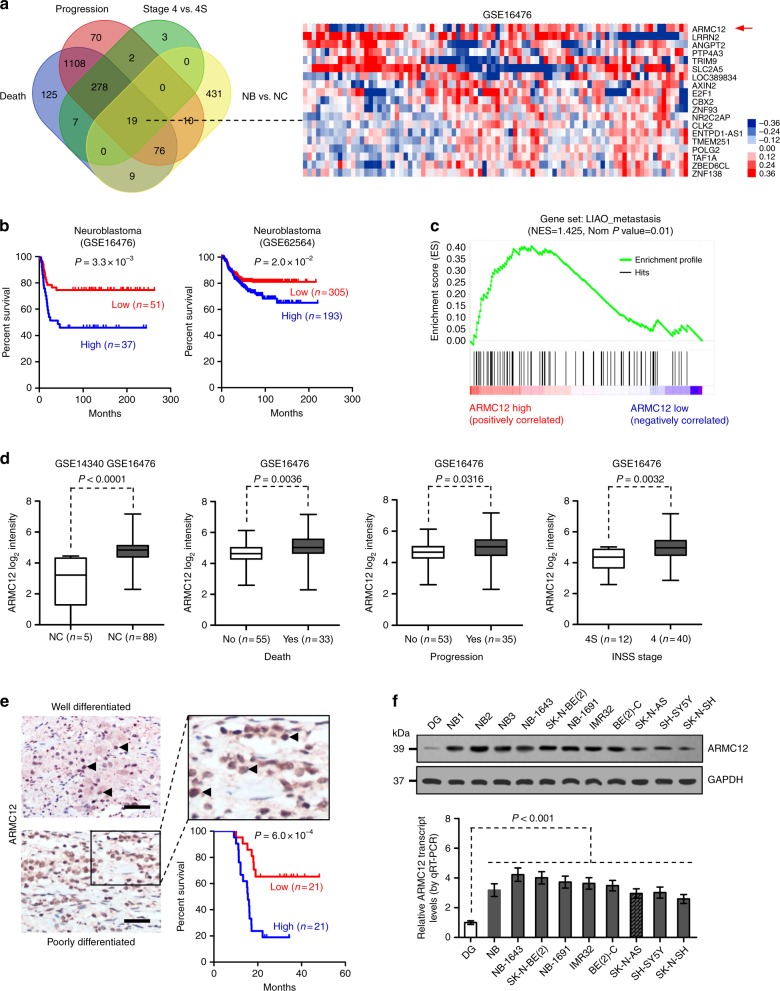
Identification of *ARMC12* as an ARM member associated with NB progression. **a** Venn diagram (left panel) and heatmap (right panel) revealing the identification of elevated genes associated with poor outcome of NB (*P* < 0.01, unpaired two-sided *t* test, FDR < 0.05) using public datasets of neural crest (NC, GSE14340) and 88 NB cases (GSE16476) with various status of death, progression, and INSS stages. **b** Kaplan−Meier curves indicating the survival of 88 (GSE16476, cutoff value = 31.1) and 498 (GSE62564, cutoff value = 4.371) NB patients with high or low *ARMC12* expression. **c** Gene set enrichment analysis of *ARMC12*-correlated genes in 88 NB tissues derived from publically available dataset (GSE16476). NES normalized enrichment score. Nom normalized. **d** Mining of public datasets (GSE14340 and GSE16476) revealing the differential expression of *ARMC12* transcript in NC (*n* = 5) and NB tissues (*n* *=* 88) with different status of death, progression, and INSS stages. **e** Representative immunohistochemical staining (upper and lower left panels) showing the expression of ARMC12 in the tumor cells of NB specimens (arrowheads, brown). Scale bars: 100 μm. Kaplan–Meier survival curve (lower right panel) of 42 NB patients with high or low ARMC12 immunostaining. **f** Western blot (upper panel) and real-time qRT-PCR (lower panel, normalized to GAPDH) indicating the expression of *ARMC12* in normal dorsal ganglia (DG, *n* *=* 21), NB tissues (*n* = 42), and cultured NB cell lines (*n* *=* 6 per cell line) with [NB-1643, SK-N-BE(2), NB-1691, IMR32, BE(2)-C] or without *MYCN* amplification (SK-N-AS, SH-SY5Y, SK-N-SH). Fisher’s exact test for overlapping analysis in **a**; log-rank test in **b** and **e**; unpaired two-sided *t* test in **d**; one-way ANOVA with Bonferroni’s multiple comparison test in **f**. Bars are means and whiskers (min to max) in **d**. Data are shown as mean ± s.e.m. (error bars) and representative of three independent experiments in **f**

To investigate the genes crucial for NB progression and patients’ outcome, we first analyzed the publicly available datasets of 88 patients (GSE16476)^14^ and neural crest (NC, GSE14340) derived from Gene Expression Omnibus (GEO). We identified 545 elevated genes associated with poor outcome in NB tissues when compared to NC (*P* < 0.01, unpaired *t* test, false discovery rate (FDR) < 0.05, Fig. 1a). In addition, 1622, 1563, and 309 highly expressed genes associated with poor outcome (*P* < 0.01, unpaired *t* test, FDR < 0.05) were noted in NB specimens with death, clinical progression, or advanced International Neuroblastoma Staging System (INSS) stages, when compared to those without these features, respectively (Fig. 1a). Based on overlapping analysis of these genes, we focused on 19 genes that were consistently associated with development, death, progression, and advanced stages of NB (*P* < 0.001, Fisher’s exact test, Fig. 1a). Among these genes, log-rank test of 88 (GSE16476) and 498 (GSE62564)^15^ NB cases revealed that patients with high levels of *ARMC12*, an ARM member highly conserved among human and primate species (Supplementary Fig. 1a), had poorer survival than those with low expression levels (*P* = 3.3×10^−3^ and *P* = 2.0×10^−2^, log-rank test, Fig. 1b). In addition, gene set enrichment analysis (GSEA) of all *ARMC12*-correlated genes in 88 specimens revealed their close relationship with cancer metastasis (Fig. 1c). Consistently, mining of publicly available microarray datasets (GSE14340 and GSE16476) revealed that *ARMC12* was overexpressed in NB tissues, when compared to that in NC (*P* < 0.0001, unpaired *t* test), and highly expressed in NB tissues with death (*P* = 0.0036, unpaired *t* test), progression (*P* = 0.0316, unpaired *t* test), or advanced INSS stages (*P* = 0.0032, unpaired *t* test, Fig. 1d and Supplementary Table 1). However, the copy number of *ARMC12* gene was neither significantly altered in NB (Supplementary Fig. 1b) nor associated with the death, *MYCN* amplification, INSS stages, or survival of NB cases derived from Oncogenomics database (Supplementary Fig. 1b and Supplementary Fig. 1c). There were no genetic variants, including missense mutation, nonsense mutation, insertion, or deletion, of *ARMC12* gene in 563 NB cases derived from three independent genome-wide studies^14,16,17^ and Therapeutically Applicable Research to Generate Effective Treatments (TARGET) database (Supplementary Fig. 1d). Low frequency of copy number alteration (amplification or deletion, 1.52%) or genetic variation (0.53%) of *ARMC12* was detected in common human cancers (Supplementary Fig. 1d). Notably, two synonymous mutations (410T>C and 770G>A) within the coding exons of *ARMC12* gene were occasionally (3/30 and 3/30) observed in our series of 30 NB cases, when compared to healthy age-matched pediatric population (Supplementary Fig. 1e and Supplementary Table 2). Among three potential *AMRC12* transcript variants revealed by GenBank (Gene ID: 221481), the 1231-bp variant (consisting of six exons) was the mainly expressed *ARMC12* transcript within NB tissues, while two other variants of low abundance were occasionally (3/30 and 4/30) detected (Supplementary Fig. 2a and Supplementary Table 2). We further validated higher *ARMC12* levels in an independent cohort of 42 primary NB tissues. Immunohistochemical staining revealed nuclear ARMC12 expression in 32/42 (76.2%) NB tissues, which was higher in tumor cases with poor differentiation (*P* = 0.013, Pearson chi-square test), higher mitosis karyorrhexis index (MKI, *P* = 0.006, Pearson chi-square test), or advanced INSS stages (*P* = 0.008, Pearson chi-square test, Supplementary Table 3). Lower survival probability (*P* = 6.0×10^−4^, log-rank test) was noted in patients with high ARMC12 expression, when compared to those with low expression (Fig. 1e). Notably, nuclear ARMC12 immunostaining was also observed in the specimens of breast cancer, colon cancer, hepatocellular carcinoma, lung cancer, pancreas cancer, prostate cancer, renal cancer, and gastric cancer, but not in their normal counterparts (Supplementary Fig. 2b). Importantly, patients with high *ARMC12* levels had less survival possibility in many types of human cancers (Supplementary Fig. 3). Western blot and real-time quantitative RT-PCR (qRT-PCR) revealed higher *ARMC12* levels in NB tissues and cell lines, than those of normal dorsal ganglia (Fig. 1f). Moreover, the transcript levels of *ARMC12* were higher in NB tissues with *MYCN* amplification (*P* = 0.0007, unpaired *t* test), advanced INSS stages (*P* = 0.0159, unpaired *t* test), or poor differentiation (*P* = 0.0024, unpaired *t* test, Supplementary Fig. 4). These data suggested that *ARMC12*, an ARM member, was associated with NB progression.

### *ARMC12* facilitates the growth and aggressiveness of NB cells

**Fig. 2:**
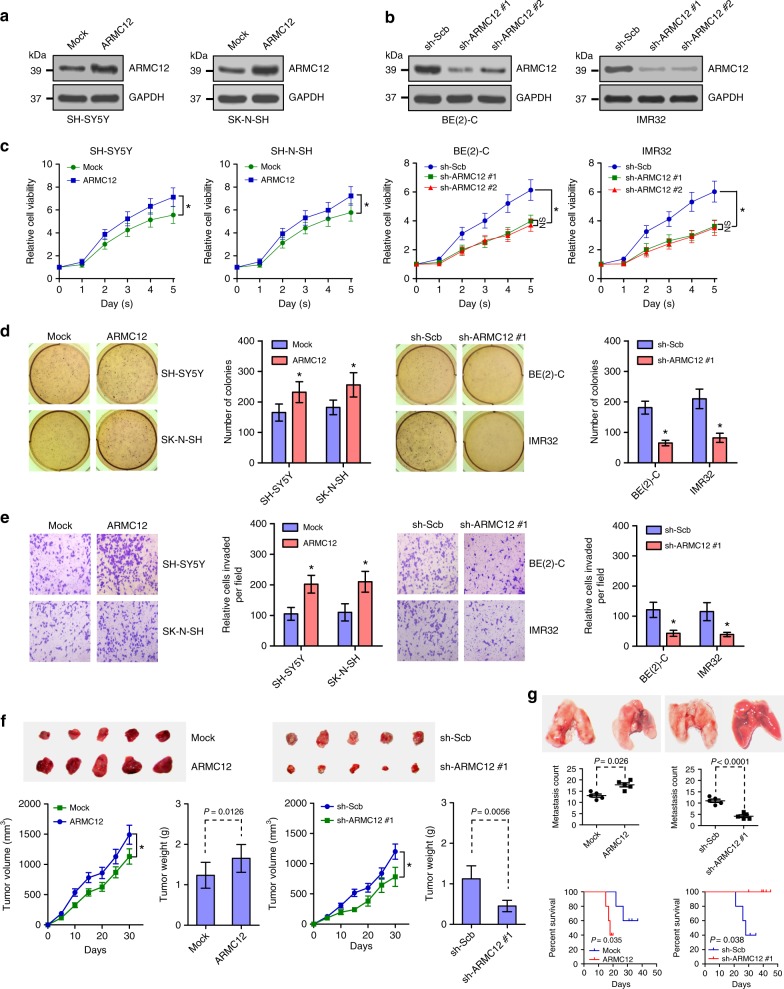
*ARMC12* facilitates the growth and aggressiveness of NB cells in vitro and in vivo. **a**, **b** Western blot assay indicating the expression of ARMC12 in NB cells stably transfected with empty vector (mock), *ARMC12*, scramble shRNA (sh-Scb), or sh-ARMC12. **c** MTT colorimetric assay depicting the change in cell viability of NB cells stably transfected with mock, *ARMC12*, sh-Scb, or sh-ARMC12 (*n* *=* 6 per time point). **d** Representative images (left panel) and quantification (right panel) of soft agar assay showing the anchor-independent growth of NB cells stably transfected with mock, *ARMC12*, sh-Scb, or sh-ARMC12 #1 (*n* *=* 4 per group). **e** Representative images (left panel) and quantification (right panel) of matrigel invasion assay indicating the invasive capability of NB cells stably transfected with mock, *ARMC12*, sh-Scb, or sh-ARMC12 #1 (*n* *=* 4 per group). **f** Representative images (upper panel), in vivo growth curves (lower left panel), and weight at the end points (lower right panel) of xenografts formed by subcutaneous injection of SH-SY5Y and BE(2)-C cells stably transfected with mock, *ARMC12*, sh-Scb, or sh-ARMC12 #1 into the dorsal flanks of nude mice (*n* *=* 5 per group). **g** Representative images (upper panel) and quantification (middle panel) of lung metastatic colonization and Kaplan−Meier curves (lower panel) of nude mice treated with tail vein injection of SH-SY5Y and BE(2)-C cells stably transfected with mock, *ARMC12*, sh-Scb, or sh-ARMC12 #1 (*n* *=* 5 per group). *P < 0.01 vs. mock or sh-Scb. One-way ANOVA with Bonferroni’s multiple comparison test in **c**; unpaired two-sided *t* test in **c−g**; log-rank test for survival comparison in **g**. NS, not significant. Data are shown as mean ± s.e.m. (error bars) and representative of three independent experiments in **a**−**e**

Since *ARMC12* expression was associated with NB progression, we explored the functional impact of *ARMC12* overexpression or knockdown on NB cell lines representing low or high expression levels. Stable transfection of *ARMC12* led to its overexpression in SH-SY5Y and SK-N-SH cells (Fig. 2a), while depletion of *ARMC12* using two independent short hairpin RNAs (shRNAs), sh-ARMC12 #1 and sh-ARMC12 #2, in the BE(2)-C and IMR32 cell lines resulted in a dramatic decrease in *ARMC12* expression levels (Fig. 2b). MTT colorimetric studies indicated that overexpression or silencing of *ARMC12* respectively facilitated and decreased the amount of viable tumor cells, than those transfected by empty vector (mock) or scramble shRNA (sh-Scb; Fig. 2c). Soft agar and matrigel invasion assays revealed that the anchor-independent growth and invasiveness of NB cells in vitro were enhanced and inhibited following stable transfection of *ARMC12* or sh-ARMC12 #1 (Fig. 2d, e). Consistent with these findings, a significant increase or reduction in the growth of NB cells and tumor weight of their formed subcutaneous xenografts in nude mice was noted upon stable overexpression or silencing of *ARMC12*, respectively (Fig. 2f). Meanwhile, more metastatic colonies in the lung and lower survival probability were observed in nude mice that received injection of SH-SY5Y cells stably overexpressing *ARMC12* via the tail vein (Fig. 2g). Conversely, fewer metastatic colonies in the lung and greater survival probability were noted in nude mice treated by tail vein administration of BE(2)-C cells with stable knockdown of *ARMC12* (Fig. 2g). Together, these results suggested that *ARMC12* facilitated the growth and aggressiveness of NB cell lines.

### ARMC12 interacts with RBBP4 to facilitate the EZH2 activity

**Fig. 3:**
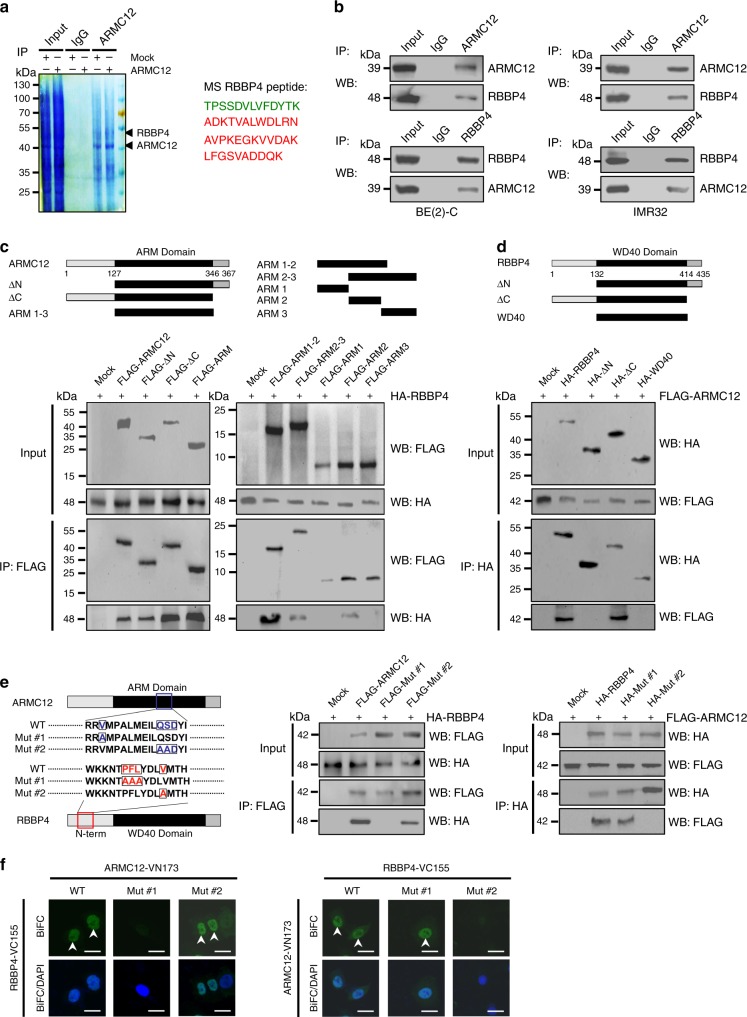
ARMC12 interacts with RBBP4 protein in NB cells. **a** Co-IP, Commassie blue staining (left panel) and mass spectrometry (MS) assay (right panel) showing the identification of ARMC12-binding protein in SH-SY5Y cells stably transfected with empty vector (mock) or *ARMC12*. **b** Co-IP and western blot assays indicating the endogenous interaction between ARMC12 and RBBP4 protein in BE(2)-C and IMR32 cells. The immunoglobulin G (IgG)-bound protein was taken as negative control. **c** Co-IP and western blot assays (lower panel) depicting the interaction between ARMC12 and RBBP4 protein in SH-SY5Y cells transfected with mock, FLAG-tagged *ARMC12* truncates (upper panel), and HA-tagged *RBBP4*. **d** Co-IP and western blot assays (lower panel) showing the interaction between ARMC12 and RBBP4 protein in SH-SY5Y cells transfected with mock, HA-tagged *RBBP4* truncates (upper panel), and FLAG-tagged *ARMC12*. **e**, **f** Co-IP and western blot (**e**, middle and right panels), and BiFC (**f**, arrowheads) assays indicating the interaction between ARMC12 and RBBP4 protein in SH-SY5Y cells transfected with wild-type or mutant (**e**, left panel) FLAG-tagged *ARMC12* and HA-tagged *RBBP4*. Scale bars: 10 μm. Data are representative of three independent experiments

To determine the interacting partner of ARMC12, mass spectrometry analysis was undertaken to identify the protein pulled down by ARMC12 antibody from extracts of SH-SY5Y and SK-N-SH cells with stable transfection of empty vector (mock) or *ARMC12*. The differential protein interacting with ARMC12 was determined by comparing the mock and *ARMC12* transfection groups. Overlapping analysis of the differential protein in each cell line indicated that RBBP4, a component of PRC2^18^, was the consistently enriched protein (with 47 detectable peptides) induced by *AMRC12* overexpression (Fig. 3a and Supplementary Table 4). Endogenous physical interaction between ARMC12 and RBBP4 protein was validated by co-immunoprecipitation (co-IP) followed by western blotting in BE(2)-C and IMR32 cells (Fig. 3b). To address their interaction domains, FLAG-tagged *ARMC12* truncation constructs were cotransfected with hemagglutinin (HA)-tagged *RBBP4* vector into SH-SY5Y cells. Co-IP and western blot assays revealed that the ARM domain [128–346 amino acid (aa)], especially ARM2 (206–245 aa), but not N-terminus (1–127 aa) or C-terminus (347–367 aa), of ARMC12 protein was crucial for its binding to RBBP4 (Fig. 3c). Meanwhile, the N-terminus (1–132 aa), but not WD40 domain (133–414 aa) or C-terminus (415–435 aa), of HA-tagged RBBP4 protein was crucial for its binding to ARMC12 (Fig. 3d). We further investigated the amino acids crucial for the interaction between ARMC12 and RBBP4 by mutagenesis assay. Mutation of Valine_217_ residue within the ARM2 domain of ARMC12 or Valine_35_ residue within the N-terminus of RBBP4 abolished their interaction (Fig. 3e, f). Notably, due to similar molecular weight and ARM2 domain (Supplementary Fig. 5a and Supplementary Fig. 5b), FLAG-tagged protein of two *ARMC12* variants with low abundance was also able to interact with RBBP4 protein in SH-SY5Y cells (Supplementary Fig. 5c), and facilitate the anchor-independent growth and invasion of NB cells (Supplementary Fig. 5d and Supplementary Fig. 5e).

**Fig. 4:**
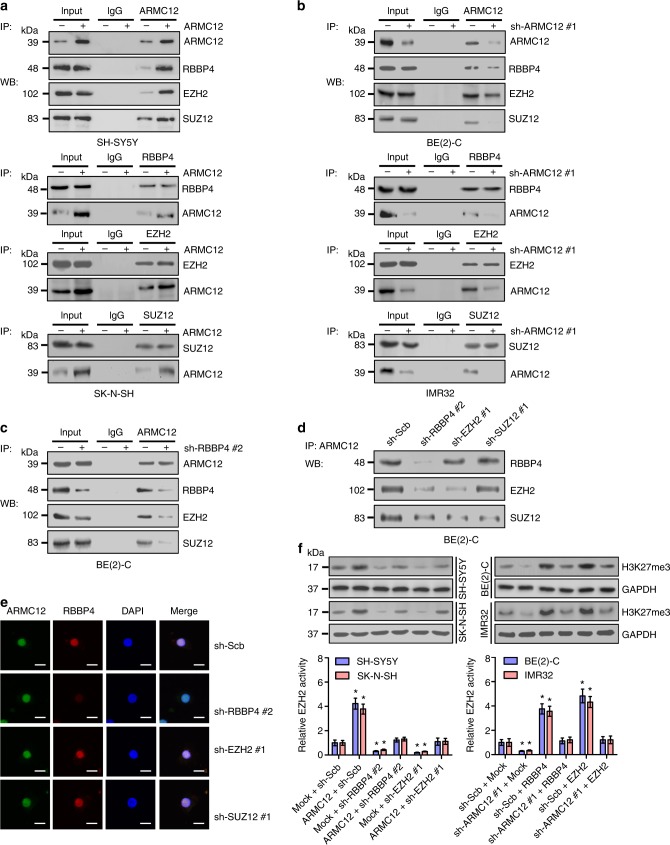
*ARMC12* facilitates the PRC2 complex formation and EZH2 activity. **a**, **b** Co-IP and western blot indicating the interaction of ARMC12 with RBBP4, EZH2, or SUZ12 in SH-SY5Y and BE(2)-C cells stably transfected with empty vector (mock), *ARMC12*, scramble shRNA (sh-Scb), or sh-ARMC12 #1. **c** Co-IP and western blot showing the interaction of ARMC12 with RBBP4, EZH2, or SUZ12 in BE(2)-C cells stably transfected with sh-Scb or sh-RBBP4 #2. **d** Co-IP and western blot indicating the interaction between ARMC12 and RBBP4 in BE(2)-C cells stably transfected with sh-Scb, sh-RBBP4 #2, sh-EZH2 #1, or sh-SUZ12 #1. **e** Immunofluorescence confocal images showing the interaction between ARMC12 and RBBP4 in BE(2)-C cells stably transfected with sh-Scb, sh-RBBP4 #2, sh-EZH2 #1, or sh-SUZ12 #1. Scale bars: 10 μm. **f** Western blot (upper panel) and chemiluminescent assay (lower panel, *n* *=* 4 per group) indicating the H3K27me3 levels and EZH2 activity in NB cells stably transfected with mock, *ARMC12*, *RBBP4*, *EZH2*, sh-Scb, sh-ARMC12 #1, sh-RBBP4 #2, or sh-EZH2 #1. *P < 0.01 vs. mock + sh-Scb (unpaired two-sided *t* test for two groups in **f**). Data are shown as mean ± s.e.m. (error bars) and representative of three independent experiments

Since previous studies indicate that RBBP4 is one component of PRC2^18^, we further investigated whether ARMC12 affected the formation of PRC2 complex. Co-IP and western blot assays revealed that ectopic expression or knockdown of *ARMC12* facilitated and repressed the interaction of ARMC12 with RBBP4, EZH2, or suppressor of zeste 12 (SUZ12) in SH-SY5Y, SK-N-SH, BE(2)-C, and IMR32 cells, respectively (Fig. 4a, b). Knockdown of *RBBP4* attenuated the interaction between ARMC12 and EZH2 or SUZ12 in BE(2)-C cells (Fig. 4c−e, and Supplementary Fig. 6). However, silencing of either *EZH2* or *SUZ12* did not affect the physical interaction between ARMC12 and RBBP4 (Fig. 4d, e, and Supplementary Fig. 6). Notably, the activity of EZH2 and expression levels of histone H3 lysine 27 trimethylation (H3K27me3) were significantly increased and reduced in *ARMC12* stable overexpressing or silencing NB cells, respectively (Fig. 4f). Consistently, a positive correlation of ARMC12 expression with H3K27me3 levels (*R* = 0.526, *P* < 0.001, Pearson’s product-moment correlation analysis) was detected in 42 NB specimens (Supplementary Fig. 7a and Supplementary Table 5). In addition, silencing of *RBBP4* or *EZH2* prevented the elevated EZH2 activity and H3K27me3 levels induced by *ARMC12* overexpression (Fig. 4f), while transfection of *RBBP4* or *EZH2* rescued the decreased EZH2 activity and H3K27me3 levels in NB cells with stable silencing of *ARMC12* (Fig. 4f). Taken together, these data suggested that ARMC12 interacted with RBBP4 protein to facilitate PRC2 complex formation and EZH2 activity in NB cells.

### *ARMC12* inhibits the expression of PRC2 target genes

**Fig. 5:**
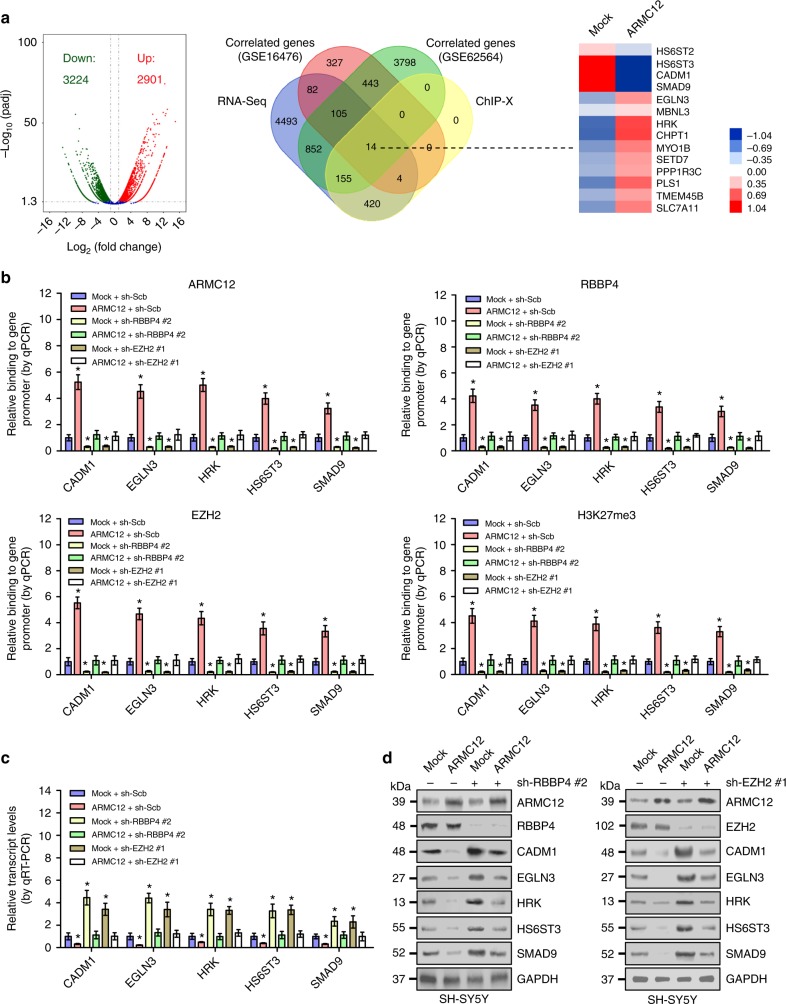
Ectopic expression of *ARMC12* represses the expression of PRC2 downstream tumor suppressive genes in NB cells. **a** Volcano plots (left panel), Venn diagram (middle panel), and heatmap (right panel) revealing the alteration of gene expression (fold change > 2.0, FDR < 0.05) in SH-SY5Y cells stably transfected with empty vector (mock) or *ARMC12*. Red indicates high expression, and blue indicates low expression in heatmap. **b** ChIP and qPCR indicating the enrichment of ARMC12, RBBP4, EZH2, or H3K27me3 (normalized to input) on target gene promoters in SH-SY5Y cells stably transfected with mock or *ARMC12*, and those cotransfected with scramble shRNA (sh-Scb), sh-RBBP4 #2, or sh-EZH2 #1 (*n* = 5 per group). **c**, **d** Real-time qRT-PCR (**c**, *n* *=* 5 per group) and western blot (**d**) assays showing the transcript and protein levels of target genes (normalized to GAPDH) in SH-SY5Y cells stably transfected with mock or *ARMC12*, and those cotransfected with sh-Scb, sh-RBBP4 #2, or sh-EZH2 #1. *P < 0.01 vs. mock + sh-Scb. Fisher’s exact test for overlapping analysis in **a**; unpaired two-sided *t* test for two groups in **b** and **c**. Data are shown as mean ± s.e.m. (error bars) and representative of three independent experiments in **b**−**d**

To determine the target genes of *ARMC12* and understand its oncogenic roles in tumorigenesis, we performed the RNA sequencing (RNA-seq) analysis to screen the differentially expressed genes with total RNA from SH-SY5Y cells stably transfected with empty vector (mock) or *ARMC12*. There were 6125 genes, including 2901 upregulated and 3224 downregulated ones, that showed differential expression (fold change > 2.0, FDR < 0.05) upon *ARMC12* overexpression (Fig. 5a). Further overlapping analysis with *ARMC12*-correlated genes in public datasets (GSE16476 and GSE62564) and PRC2 downstream targets in ChIP-X database^19^ (*P* < 0.001, Fisher’s exact test) revealed that 14 downstream genes were significantly regulated by ARMC12 (Fig. 5a). Mining of microarray (GSE16476) and RNA-seq (GSE62564) datasets indicated that among these target genes, the expression of cell adhesion molecule 1 (*CADM1*), egl-9 family hypoxia-inducible factor 3 (*EGLN3*), harakiri BCL2-interacting protein (*HRK*), heparan sulfate 6-O-sulfotransferase 3 (*HS6ST3*), and SMAD family member 9 (*SMAD9*) was significantly associated with favorable outcome of patients (Supplementary Fig. 7b).

**Fig. 6:**
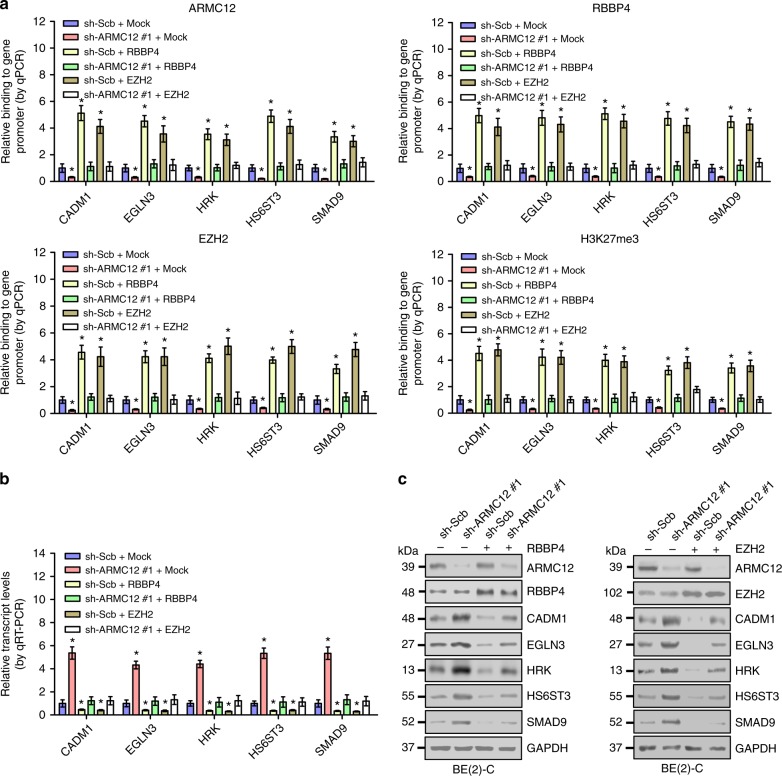
Knockdown of *ARMC12* increases the expression of PRC2 downstream tumor suppressive genes in NB cells. **a** ChIP and qPCR indicating the enrichment of ARMC12, RBBP4, EZH2, or H3K27me3 (normalized to input) on target gene promoters in BE(2)-C cells stably transfected with scramble shRNA (sh-Scb) or sh-ARMC12 #1, and those cotransfected with *RBBP4* or *EZH2* (*n* *=* 5 per group). **b**, **c** Real-time qRT-PCR (**b**, *n* *=* 5 per group) and western blot (**c**) assays showing the transcript and protein levels of target genes (normalized to GAPDH) in BE(2)-C cells stably transfected with sh-Scb or sh-ARMC12 #1, and those cotransfected with *RBBP4* or *EZH2*. *P < 0.01 vs. sh-Scb + mock (unpaired two-sided *t* test for two groups in **a** and **b**). Data are shown as mean ± s.e.m. (error bars) and representative of three independent experiments

Since above evidence has revealed the roles of ARMC12 in facilitating PRC2 complex formation and EZH2 activity, and combining the essential roles of EZH2 and H3K27me3 in transcriptional repression^20–22^, we further investigated the impact of *AMRC12* on the expression of these PRC2 target genes. Chromatin immunoprecipitation (ChIP) and quantitative PCR (qPCR) assays revealed that ectopic expression of *ARMC12* facilitated the binding of ARMC12, RBBP4, EZH2, and H3K27me3 to the promoter regions of *CADM1*, *EGLN3*, *HRK*, *HS6ST3*, and *SMAD9*, respectively (Fig. 5b). In addition, overexpression of *ARMC12* suppressed the transcriptional and protein levels of these target genes in NB cells (Fig. 5c, d). Conversely, knockdown of *ARMC12* reduced the enrichment of ARMC12, RBBP4, EZH2, and H3K27me3 on target gene promoters (Fig. 6a), and increased the expression of *CADM1*, *EGLN3*, *HRK*, *HS6ST3*, and *SMAD9* in NB cells (Fig. 6b, c). Importantly, rescue experiments indicated that knockdown or ectopic expression of *RBBP4* or *EZH2* abolished the change in promoter enrichment and expression of *CADM1*, *EGLN3*, *HRK*, *HS6ST3*, and *SMAD9* induced by overexpression or knockdown of *ARMC12*, respectively (Figs. 5b−d, 6a−c). Meanwhile, knockdown or ectopic expression of *RBBP4* or *EZH2* prevented the SH-SY5Y and BE(2)-C cells from increased and decreased growth and invasion induced by overexpression or silencing of *ARMC12*, respectively (Supplementary Fig. 8a, Supplementary Fig. 8b, Supplementary Fig. 8c, and Supplementary Fig. 8d). These results indicated that *ARMC12* suppressed the expression of PRC2 downstream tumor suppressive targets in NB cells.

### Therapeutic peptide blocking the ARMC12-RBBP4 interaction

**Fig. 7:**
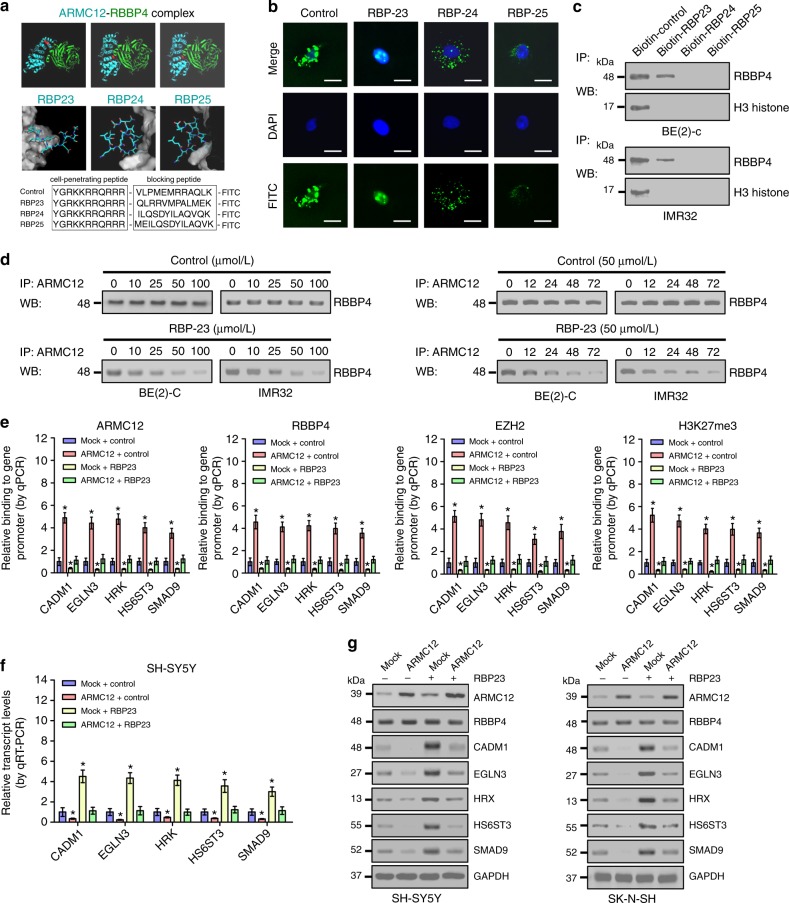
Inhibitory peptide blocks the interaction between ARMC12 and RBBP4 in NB cells. **a** Predicted structure and sequences of inhibitory peptides for blocking the interaction between ARMC12 and RBBP4. **b** Confocal images showing the distribution of synthesized inhibitory peptides within cultured BE(2)-C cells. Scale bars: 10 μm. **c** Biotin-labeled peptide pull-down and western blot assays indicating the binding of inhibitory peptides (50 μmol L^−1^) to RBBP4 protein within cell lysates. **d** Co-IP and western blot revealing the interaction between ARMC12 and RBBP4 in NB cells treated with different doses of control or inhibitory (RBP23) peptide for 24 h, or 50 μmol L^−1^ control peptide or RBP23 for time points as indicated. **e** ChIP and qPCR showing the enrichment of ARMC12, RBBP4, EZH2, or H3K27me3 (normalized to input) on target gene promoters in SH-SY5Y cells stably transfected with mock or *ARMC12*, and those treated with inhibitory peptide RBP23 (50 μmol L^−1^) for 24 h (*n* = 5 per group). **f**, **g** Real-time qRT-PCR (**f**, *n* *=* 5 per group) and western blot (**g**) indicating the expression of target genes (normalized to GAPDH) in SH-SY5Y cells and SK-N-SH stably transfected with mock or *ARMC12*, and those treated with inhibitory peptide RBP23 (50 μmol L^−1^) for 24 h. *P < 0.01 vs. mock + control (unpaired two-sided *t* test for two groups in **e** and **f**). Data are shown as mean ± s.e.m. (error bars) and representative of three independent experiments

To further investigate the functional interplay of ARMC12 and RBBP4 during the aggressiveness of NB cells, we designed cell-penetrating inhibitory peptides with 23, 24, and 25 amino acids in length, named as RBBP4 binding peptide 23 (RBP23), RBP24, and RBP25 (Fig. 7a), that might potentially block the interaction between ARMC12 and RBBP4^23^. Treatment of NB cells with RBP23, but not with RBP24 or RBP25, resulted in obvious aggregation of peptide within the nucleus (Fig. 7b). Pull-down assay using biotin-labeled peptides indicated that RBP23, but not RBP24 or RBP25, was able to bind to endogenous RBBP4 protein in the lysates of BE(2)-C and IMR32 cells (Fig. 7c). Meanwhile, RBP23 treatment of BE(2)-C and IMR32 cells abolished the endogenous interaction between ARMC12 and RBBP4 in a dose- and time-dependent manner (Fig. 7d). In addition, treatment of SH-SY5Y cells with RBP23 abolished the increased enrichment of ARMC12, RBBP4, EZH2, and H3K27me3 on the promoter regions of *CADM1*, *EGLN3*, *HRK*, *HS6ST3*, and *SMAD9* induced by *ARMC12* overexpression (Fig. 7e). Accordingly, the repressed expression of these genes in *ARMC12* overexpressing SH-SY5Y and SK-N-SH cells was rescued by treatment with RBP23 (Fig. 7f, g).

**Fig. 8:**
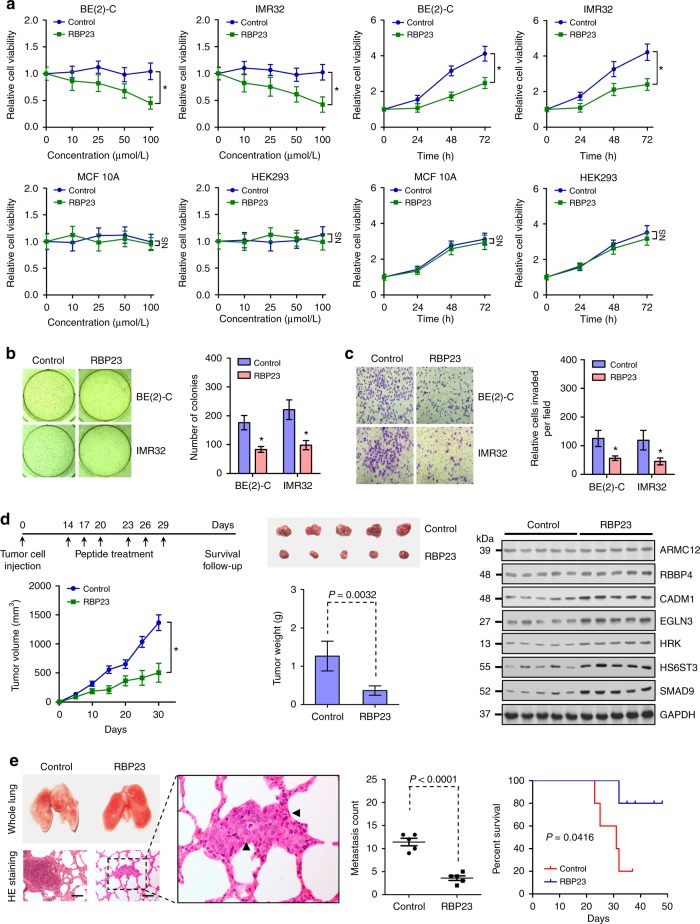
Inhibitory peptide suppresses the tumorigenesis and aggressiveness of NB cells in vitro and in vivo. **a** MTT colorimetric assay depicting the change in viability of NB cells, nontransformed MCF 10A cells, and transformed HEK293 cells treated with different doses of control or inhibitory (RBP23) peptide for 24 h, or 50 μmol L^−1^ control peptide or RBP23 for time points as indicated (*n* *=* 6 per group). **b**, **c** Representative images (left panel) and quantification (right panel) of soft agar (**b**) and transwell matrigel invasion (**c**) assays showing the anchor-independent growth and invasion capability of viable NB cells pretreated with control peptide or RBP23 (10 μmol L^−1^) for 24 h (*n* *=* 4 per group). **d** The in vivo growth curve (left lower panel), representative images (middle upper panel), tumor weight at the end points (middle lower panel), and gene expression detected by western blot (right panel) of xenografts formed by subcutaneous injection of BE(2)-C cells in nude mice (*n* *=* 5 per group) that subsequently treated with intratumoral injection of control peptide or RBP23 (3 mg kg^−1^) as indicated (left upper panel). **e** Representative images (left panel) and quantification (middle panel) of lung metastatic colonization and Kaplan−Meier curves (right panel) of nude mice (*n* *=* 5 per group) treated with tail vein injection of BE(2)-C cells and subsequent administration of control peptide or RBP23 (3 mg kg^−1^) as indicated. Scale bars: 100 μm. *P < 0.01 vs. control. Unpaired two-sided *t* test in **a−e**; log-rank test in **e**. NS, not significant. Data are shown as mean ± s.e.m. (error bars) and representative of three independent experiments in **a**−**c**

Then, we investigated the therapeutic efficiency of inhibitory peptide on the tumorigenesis and aggressiveness of NB cells in vitro and in vivo. Administration of RBP23 suppressed the viability of BE(2)-C and IMR32 cells in a dose- and time-dependent manner (Fig. 8a). In contrast, RBP23 treatment resulted in no significant alteration in the viability of MCF 10A and HEK293, nontransformed and transformed normal cells lacking *ARMC12* expression (Supplementary Fig. 5a and Supplementary Fig. 8e), when compared to those treated with control peptide (Fig. 8a). Moreover, the anchor-independent growth and invasiveness of viable NB cells were significantly inhibited by administration of RBP23 (Fig. 8b, c). Then, the therapeutic effects of RBP23 on the growth of subcutaneous xenografts and survival of blindly randomized nude mice were investigated in vivo (Fig. 8d). Intratumoral administration of RBP23 led to significant reduction in the volume and weight of subcutaneous xenografts established by injection of BE(2)-C cells, than those in control peptide treatment group (Fig. 8d). The levels of *ARMC12* downstream tumor suppressive targets were obviously enhanced after intratumoral injection of RBP23 (Fig. 8d). In metastasis assay, administration of RBP23 via the tail vein significantly reduced the lung metastatic colonies formed by BE(2)-C cells in athymic nude mice (Fig. 8e). Log-rank test indicated that administration of RBP23 obviously increased the survival duration of nude mice (Fig. 8e). Collectively, these results indicated that inhibitory peptide blocking the interaction between ARMC12 and RBBP4 suppressed the in vitro and in vivo growth and aggressiveness of NB cells.

### High *RBBP4* expression is associated with poor outcome of NB

To explore the *RBBP4* expression in NB, fresh and paraffin-embedded specimens were collected from 42 primary cases. Immunohistochemical staining indicated the nuclear RBBP4 expression in tumor cells (Supplementary Fig. 9a). The RBBP4 immunostaining was noted in 30/42 (71.4%) NB tissues, and higher in specimens with poor differentiation (*P* = 0.011, Pearson chi-square test), higher MKI (*P* = 0.016, Pearson chi-square test), or advanced INSS stages (*P* < 0.001, Pearson chi-square test, Supplementary Table 6). Western blot and real-time qRT-PCR assays revealed higher *RBBP4* levels in NB tissues and cell lines, than those in normal dorsal ganglia (Supplementary Fig. 9b). Analysis of publicly available datasets (GSE14340 and GSE16476) indicated that the *RBBP4* transcript levels were higher in NB than those in NC (*P* < 0.0001, unpaired *t* test), and highly expressed in NB cases with death (*P* = 0.0354, unpaired *t* test), progression (*P* = 0.0403, unpaired *t* test), or advanced INSS stages (*P* = 0.0062, unpaired *t* test, Supplementary Fig. 9c). In an independent series of 42 primary NB cases, the transcript levels of *RBBP4* were higher in NB specimens with *MYCN* amplification (*P* = 0.0149, unpaired *t* test), advanced INSS stages (*P* = 0.0002, unpaired *t* test), or poor differentiation (*P* = 0.0006, unpaired *t* test, Supplementary Fig. 9d), without significant difference in NB patients of varied age groups (*P* = 0.9693, unpaired *t* test, Supplementary Fig. 9d). Log-rank test of 42 NB cases revealed that patients with high *RBBP4* levels had poorer survival probability (*P* = 5.0×10^−4^, log-rank test) than those with low levels (Supplementary Fig. 9e), which was consistent with the findings from public dataset (GSE16476, Supplementary Fig. 9e). These data suggested that high *RBBP4* levels were associated with poor outcome of NB.

## Discussion

The ARM motif repeats were initially identified from the *armadillo* gene in *Drosophila*^24^. Since then, a series of studies have shown that ARM repeats are well conserved through eukaryotes, and many homologous motifs are discovered in armadillo-repeat-containing proteins^25–27^. Members of the ARM protein family interact with numerous different binding partners through their ARM repeat domain, and exert diverse essential functions in cell adhesion, cell−cell contact, signal transduction, and tumorigenesis^25–27^. For example, armadillo repeat-containing X-linked 3 (ARMCX3) regulates the subcellular localization and transcriptional activity of sex determining region Y box 10 (SOX10) through physical interaction via the ARM repeat domain^28^. In this study, we discover *ARMC12* as an ARM member associated with NB progression and poor outcome of patients. Our initial evidence also reveals the significant correlation of *ARMC12* levels with the outcome of other cancer types. In addition, *ARMC12* exerts oncogenic functions, such as promotion of the growth and aggressiveness of NB cells. As a nuclear protein, ARMC12 interacts with RBBP4 protein via the ARM domain to increase the formation of PRC2 complex and facilitate the EZH2 activity, which represses the transcription of downstream tumor suppressive genes (Supplementary Fig. 9f). Notably, our findings indicate that despite low abundance, two other *ARMC12* variants may also exert similar oncogenic functions in tumorigenesis and aggressiveness, which warrants further investigation. Meanwhile, inhibitory peptide blocking the ARMC12−RBBP4 interaction is able to suppress the biological features of tumor cells, but not detrimental to normal cells lacking the endogenous *ARMC12* expression. The discovery of such an ARM repeat protein represents a promising step for the therapeutic intervention against NB.

As a ubiquitously expressed nuclear protein of WD40 family, RBBP4 was initially identified based on its interaction with the carboxyl terminus of retinoblastoma protein^29^. Subsequent studies show that RBBP4 protein is a component of various complexes involved in chromatin assembly, remodeling, and nucleosome modification, including Sin3^30^, PRC2^18^, nucleosome remodeling factor^31^, histone acetyltransferase 1 (HAT1)^32^, and chromatin assembly factor 1 (CAF1)^33^, and plays very different roles in each setting. RBBP4 serves as a targeting molecule to bring histone deacetylases, HAT1, or CAF1 to their histone substrates^32,33^. It has been indicated that *RBBP4* expression is elevated and correlated with the malignant phenotypes in many types of human tumors, such as lung cancer^34^, liver cancer^35^, thyroid carcinomas^36^, acute myelocytic leukemia^37^, and acute lymphoblastic leukemia^37^. Dysregulation of *RBBP4* is linked to carcinogenesis in breast tissues^38,39^, and controls the human papillomavirus type 16 transforming activity in cervical carcinogenesis through regulating the expression of tumor suppressive (retinoblastoma and *p53*) or oncogenic (*cyclin D1* and *c-Myc*) genes^40^. In thyroid cancer, RBBP4 promotes the growth potential of cancer cells through influencing the functions of genes controlling cell cycle progression^36^. In contrast, overexpression of *RBBP4* induces cell cycle arrest and apoptosis in irradiated cervical cancer cells, which is related to upregulation of *p53*, retinoblastoma protein, and *caspase-8*^41^. As a radiosensitive gene, RBBP4 induces the radiosensitization in breast cancer and melanoma cell lines^42^. These findings suggest the oncogenic or tumor suppressive roles of *RBBP4* in a context-dependent manner. In this study, our evidence indicated that *RBBP4* was associated with unfavorable outcome of NB, and facilitated the growth and aggressiveness of NB cells, indicating its oncogenic functions in NB progression.

It has been established that PRC2 is the major methyltransferase for H3K27 methylation, and high levels of H3K27me3 in gene promoter region generally correlate with transcriptional repression in animals and plants^20–22^. The PRC2 complex is consisted of four core subunits, including EZH2, SUZ12, embryonic ectoderm development (EED), and RBBP4^43^. The catalytic activity of PRC2 is conferred by EZH2, whereas EED and SUZ12 are necessary for the integrity and activity of PRC2 in H3K27 methylation^20–22^. Meanwhile, RBBP4 is critical for H3K27me3 only at subtelomeric domains^44^. In this study, we identify that ARMC12 increases the EZH2 activity and H3K27me3 levels in an RBBP4-dependent manner, and facilitates the enrichment of PRC2 and H3K27me3 on gene promoters, resulting in transcriptional repression of tumor suppressors affecting the proliferation, invasion, and metastasis of tumor cells, such as *CADM1*^45^, *EGLN3*^46^, *HRK*^47^, *HS6ST3*^48^, and *SMAD9*^49^. We note that ectopic expression of *ARMC12* rescues the decreased H3K27me3 enrichment on target gene promoters induced by *EZH2* knockdown in NB cells, suggesting that the binding of AMRC12 to RBBP4 may result in structure alteration of PRC2 that facilitates the activity of EZH2 expressed at relatively low levels, which warrants further investigation. Since knockdown of *RBBP4* abolishes the alteration in aggressive behaviors of NB cells mediated by *ARMC12*, our evidence indicates that the oncogenic roles of ARMC12 are mediated, at least in part, via interacting with RBBP4 protein.

In summary, we have demonstrated that *ARMC12* is highly expressed and associated with poor survival of NB. ARMC12 physically binds to RBBP4 to increase the PRC2 complex formation and EZH2 activity, leading to repressed gene expression of downstream tumor suppressive targets associated with NB progression. Blocking the interaction between ARMC12 and RBBP4 via administration of cell-penetrating inhibitory peptide suppresses the in vitro and in vivo growth, invasion, and metastasis of NB cells. Due to lack of association with copy number alteration or limited number of cases, the regulatory mechanisms and genetic variation of *ARMC12* in NB remain to be determined. Meanwhile, the roles and prognostic values of *ARMC12* and *RBBP4* in tumorigenesis and aggressiveness remain to be validated using transgenic or knockout animal models. This study is helpful in extending the knowledge regarding genes crucial for tumor progression, and indicates that *ARMC12* and *RBBP4* are valuable as potential targets for the treatment of NB.

## Methods

### Cell lines

Human NB cell lines with *MYCN* amplification (NB-1643, NB-1691, SK-N-BE(2) (CRL-2271), IMR32 (CCL-127), and BE(2)-C (CRL-2268)) or without *MYCN* amplification (SH-SY5Y (CRL-2266), SK-N-SH (HTB-11), and SK-N-AS (CRL-2137)), nontransformed mammary epithelial MCF 10A (CRL-10317) and transformed embryonic kidney HEK293 (CRL-1573) cells were obtained from Pediatric Oncology Group Cell Bank (Lubbock, TX) and American Type Culture Collection (Rockville, MD), authenticated by short tandem repeat profiling, and used within 6 months after resuscitation of frozen aliquots. Mycoplasma contamination was regularly examined using Lookout Mycoplasma PCR Detection Kit (Sigma, St. Louis, MO). The NB cell lines and HEK293 cells were cultured with RPMI1640 medium containing 10% fetal bovine serum (Thermo Fisher Scientific, Inc., Waltham, MA), while MCF 10A cells were cultured in DMEM/F12 medium containing 5% horse serum (Invitrogen, Carlsbad, CA) and 20 ng m^L−1^ epidermal growth factor (Peprotech, Rocky Hill, NJ). Cells were grown at 37 °C in a humidified atmosphere of 5% CO_2_.

### Data mining of public datasets

Public GEO datasets GSE16476 and GSE14340 were established by Affymetrix Human Genome U133 Plus 2.0 Array platform (GPL570). The differentially expressed genes between two groups were screened by unpaired Student’s *t* test and correction multiple testing method. Log-rank test and correction multiple testing analyses were applied to evaluate the survival significance of each gene in NB patients.

### GSEA assay

GSEA assay was undertaken as previously reported^50^, with application of indicated gene sets. Datasets were derived from either established microarray (GSE16476) or RNA-seq (GSE62564) results.

### Overexpression and knockdown of genes

Human *ARMC12* cDNA (1104 bp), *EZH2* cDNA (2256 bp) and their truncations were obtained from NB tissues by PCR (Supplementary Table 7) and inserted into corresponding vector as indicated. The FLAG-tagged expression vectors of *ARMC12* variant 2 and 3 were prepared with GeneTailor^TM^ Site-Directed Mutagenesis System (Invitrogen), using PCR primers shown in Supplementary Table 7. Human truncations of *RBBP4* cDNA (1278 bp), a kind gift from Dr. Didier Trouche^51^, were prepared using PCR primers (Supplementary Table 7) and subcloned into pCMV-HA (Beyotime Biotechnology, Shanghai, China). Mutation of *ARMC12* or *RBBP4* was prepared with GeneTailor^TM^ Site-Directed Mutagenesis System (Invitrogen), using PCR primers shown in Supplementary Table 7. Oligonucleotides specific for shRNAs against *ARMC12*, *RBBP4*, *EZH2*, or *SUZ12* (Supplementary Table 7) were inserted into GV102 (Genechem Co., Ltd, Shanghai, China). After screening with puromycin or neomycin (Invitrogen), stable cancer cells were established.

### RNA sequencing

Total RNAs of tumor cells (1×10^6^) were extracted in accordance with the manual of TRIzol^®^ (Life Technologies, Inc., Gaithersburg, MD). Preparation of library and sequencing of transcriptome were carried out using Illumina HiSeq X Ten (Novogene Bioinformatics Technology Co., Ltd., Beijing, China). The mapping of 100-bp paired-end reads to genes was undertaken using HTSeq v0.6.0 software, while fragments per kilobase of transcript per million fragments mapped (FPKM) were also analyzed. The results of RNA sequencing have been submitted to GEO database (accession number: GSE107516).

### Real-time quantitative RT-PCR and PCR

Total RNAs were prepared by RNeasy Mini Kit (Qiagen Inc., Redwood City, CA). The Transcriptor First Strand cDNA Synthesis Kit (Roche) was applied for reverse transcription. For real-time RT-PCR, SYBR Green PCR Master Mix (Applied Biosystems, Foster City, CA) and primers (Supplementary Table 8) were applied, with transcript levels being determined by 2^−Δ^^Δ^^Ct^ method. Genomic DNA was isolated using QIAamp DNA mini kit (Qiagen Inc.). The genetic variation within exons of *ARMC12* gene was analyzed by PCR using primers (Supplementary Table 8) and Sanger sequencing.

### Western blotting

Protein of tumor cells or tissues was prepared using 1× cell lysis buffer (Promega). Western blotting was performed as described previously^52–55^, with antibodies specific for ARMC12 (ab72704, Abcam, Cambridge, MA, 1:1000 dilution), RBBP4 (ab1765, Abcam, 1:1000 dilution), EZH2 (ab191250, Abcam, 1:1000 dilution), SUZ12 (ab12073, Abcam, 1:500 dilution), H3K27me3 (ab6002, Abcam, 1:1000 dilution), HA (ab9110, Abcam, 1:1000 dilution), FLAG (ab125243, Abcam, 1:1000 dilution), CADM1 (ab3910, Abcam, 1:500 dilution), EGLN3 (ab30782, Abcam, 1:1000 dilution), HRK (ab45419, Abcam, 1:1000 dilution), HS6ST3 (SAB2101082, Sigma, 1:500 dilution), SMAD9 (ab115900, Abcam, 1:500 dilution), histone H3 (ab1791, Abcam, 1:1000 dilution), and GAPDH (ab8245, Abcam, 1:1000 dilution). Uncropped images of blots were provided in Supplementary Fig. 10.

### Co-IP and mass spectrometry analysis

Co-IP was carried out as reported previously^56^, using 10 µg of antibodies for ARMC12 (ab72704, Abcam), RBBP4 (ab1765, Abcam), EZH2 (ab191250, Abcam), SUZ12 (ab12073, Abcam), FLAG (ab125243, Abcam), and HA (ab9110, Abcam). After releasing from bead-bound complex, protein levels were measured by western blotting. Uncropped images of blots were shown in Supplementary Fig. 10. For proteomic analysis, proteins were digested by trypsin, and peptides were extracted according to Invitrogen protocol. LC-MS/MS detection was undertaken on a hybrid quadrupole-TOF LC/MS/MS mass spectrometer (TripleTOF 5600+, SCIEX, Redwood City, CA). For each scan cycle, one full-scan mass spectrum (with m/z ranging from 350 to 1500 and charge states from 2 to 5) and subsequent 40 MS/MS events were carried out. Analysis of MS/MS-acquired data was performed using ProteinPilot Software 5.0 and amino acid sequences in UniProt human protein database.

### Bimolecular fluorescence complementation (BiFC) assay

Human *ARMC12* cDNA (1104 bp) or *RBBP4* cDNA (1278 bp) was respectively subcloned into pBiFC-VN173 and pBiFC-VC155 (Addgene, Cambridge, MA). Mutation of *ARMC12* or *RBBP4* was undertaken with GeneTailor^TM^ Site-Directed Mutagenesis System (Invitrogen) and PCR primers (Supplementary Table 7). After co-transfection of recombinant constructs using Lipofectamine 3000 (Invitrogen) for 24 h, tumor cells were grown for additional 10 h at 37 °C. Under a confocal microscope, the fluorescence emission was detected (488 and 500 nm as excitation and emission wavelengths, respectively).

### Immunofluorescence co-localization assay

Cells were grown on coverslips, incubated by 5% milk for 1 h, and treated with antibodies specific for ARMC12 (ab72704, Abcam, 1:100 dilution) or RBBP4 (ab1765, Abcam, 1:100 dilution) at 4 °C overnight. Then, coverslips were treated by Alexa Fluor 488 goat anti-rabbit IgG (ab150081, Abcam, 1:1000 dilution) or Alexa Fluor 594 goat anti-rabbit IgG (ab150160, Abcam, 1:1000 dilution), and stained by 4′,6-diamidino-2-phenylindole (DAPI, 300 nmol L^−1^). The images were photographed under a microscope^52^.

### Target gene rescue experiments

To rescue the *ARMC12* knockdown-increased levels of target genes, the expression vector of full-length *RBBP4* or *EZH2* was transfected into stable cell lines. To restore the repressed expression of target genes induced by *ARMC12*, transfection of shRNAs against *RBBP4* or *EZH2* (Supplementary Table 7) into tumor cells was performed using Genesilencer Transfection Reagent (Genlantis, San Diego, CA).

### Chromatin immunoprecipitation

ChIP assay was carried out using the EZ-ChIP kit (Upstate Biotechnology, Temacula, CA)^52,56,57^. DNA was extracted and sonicated into 200-bp size. For real-time qPCR assay, SYBR Green PCR Master Mix (Applied Biosystems) and gene promoter-specific primers (Supplementary Table 8) were applied.

### EZH2 catalytic activity analysis

The histone methyltransferase (HMT) activity of EZH2 was detected using EZH2 chemiluminescent assay kit (BPS Bioscience, San Diego, CA). Briefly, S-adenosylmethionine (400 μmol L^−1^) was incubated with nuclear extracts in 1× HMT assay buffer on 96-well plates precoated with histone H3 peptide substrate for 1 h. Then, incubation of the plate with H3K27me3 antibody (at room temperature) was undertaken for 1 h. After washing with TBST buffer, the plate was incubated with horseradish peroxidase (HRP)-labeled antibody for 30 min followed by addition of HRP substrate. The chemiluminescence was measured using a luminometer (Tecan Schweiz AB, Zurich, Switzerland).

### Design and synthesis of inhibitory peptides

The inhibitory peptides for blocking the interaction between ARMC12 and RBBP4 were designed by Peptiderive server^23^. The 11 amino acid long peptide (YGRKKRRQRRR) from the Tat protein transduction domain served as a cell-penetrating peptide. Thus, inhibitory peptides were chemically synthesized by linking with biotin-labeled cell-penetrating peptide at N-terminus and conjugating with fluorescein isothiocyanate (FITC) at C-terminus. The purity of peptides (larger than 95%) was validated by reversed phase-high performance liquid chromatography assay.

### Biotin-labeled peptide pull-down assay

Cellular proteins were isolated using 1× cell lysis buffer (Promega), and incubated with biotin-labeled peptide at 4 °C overnight. Then, incubation of cell lysates with streptavidin-agarose was undertaken at 4 °C for 2 h. The beads were extensively washed, and pulled down proteins were used for sodium dodecyl sulfate polyacrylamide gel electrophoresis (SDS-PAGE) and western blot.

### Cell viability assay

Tumor cells (3×10^3^ per well, six wells per group) were seeded in 96-well plates, and cell viability was detected by MTT (Sigma) colorimetric assay^56^. All experiments were replicated for three times.

### Soft agar assay

Tumor cells (5×10^3^ per well) were mixed with 0.05% Nobel agar, and incubated on six-well plates containing solidified 0.1% Noble agar (Thermo Fisher Scientific, Inc.) for 21 days. Cellular colonies were stained with 0.5% crystal violet dye, and counted under a microscope^52,57^.

### Colony formation assay

Tumor cells (300 cells per well) were incubated on six-well plates for 21 days. Cellular colonies were stained using 0.1% crystal violet, and the number of colonies (more than 50 cells) was counted^58^.

### Matrigel invasion assay

Cellular invasion capability was measured with Matrigel matrix (BD Science, Sparks, MD)^52,54,55,57,59–61^. Briefly, starved tumor cells (1×10^5^ per well) were added to upper chamber of Transwell insert with 8.0-μm pores (Corning, New York, NY), and allowed to invade for 24 h. Invaded cells were stained with 0.1% crystal violet for 10 min, and counted under the microscope.

### In vivo tumorigenesis and aggressiveness assays

The Animal Care Committee of Tongji Medical College approved all animal studies (approval number: Y20080290), which were performed according to NIH Guidelines for the Care and Use of Laboratory Animals. Four-week-old female BALB/c nude mice were randomized blindly (*n* = 5 per group) for subcutaneous xenografts (1×10^6^ tumor cells for each mouse) and experimental metastasis (0.4×10^6^ tumor cells for each mouse) studies as previously reported^52,53,57,62^. In therapeutic studies, nude mice received the injection of tumor cells (1×10^6^ or 0.4×10^6^) at the dorsal flanks or via the tail vein, respectively. Two weeks later, intratumoral or tail vein administration of synthesized cell-penetrating peptide (ChinaPeptides, Shanghai, China) was carried out in blindly randomized nude mice as indicated. For each mouse, the alteration in volume of tumors and survival time was recorded and analyzed.

### Clinical tissues

The Institutional Review Board of Tongji Medical College approved all human specimen studies (approval number: 2011-S085). All procedures were performed in accordance with guidelines in the Declaration of Helsinki. Written informed consent was obtained from all legal guardians of the patients. All cases received no preoperative chemotherapy or other treatment. Human normal dorsal root ganglia were collected from interrupted pregnancies. Fresh tumor tissues were collected at surgery and stored at −80 °C. The demographic and clinicopathological details of 42 cases were described in Supplementary Table 3. Total RNAs of normal human dorsal ganglia were purchased from Clontech (Mountain View, CA). Peripheral blood samples were obtained from ten healthy age-matched children attending outpatient clinics for well-child visits.

### Immunohistochemistry

Immunohistochemical staining was undertaken as described previously^52,53,57,62^, with antibodies specific for ARMC12 (ab72704, Abcam, 1:200 dilution) or RBBP4 (ab1765, Abcam, 1:200 dilution). To assess the reactivity degree, ten different high power fields (×400) for each specimen were blindly evaluated. The staining intensity was evaluated on a range from 0 to 3 (0, negative; 1, weakly positive; 2, moderately positive; 3, strongly positive), while percentage of positive cells were evaluated ranging from 0 to 4 (0, negative; 1, positive in 1−25%; 2, positive in 26−50%; 3, positive in 51−75%; 4, positive in 76−100%). Based on the products of staining intensity multiplied by percentage of positive cells, the results of immunohistochemistry were classified into negative (−, 0–1), mildly (+, 2–3), moderately (++, 4–8), and strongly positive (+++, 9–12). The moderate (++) or strong (+++) reactivity was defined as high expression, while negative (−) or mild positive (+) reactivity was defined as low expression.

### Statistical analysis

The data were shown as mean ± standard error of the mean (s.e.m., represented as error bars in the graphs). The cutoff for gene expression was defined by average values. The Pearson chi-square test, analysis of variance (ANOVA) with Bonferroni’s multiple comparison test, and Student’s *t* test were applied for comparing the difference of tumor cells or tissues. Statistical significance of overlap or expression correlation was determined by Fisher’s exact test and Pearson’s product-moment correlation analysis, respectively. Log-rank assay was applied to compare the difference in survival. Two-sided statistical analysis was applied. Randomization and blinding strategy was used whenever possible. Animal cohort sizes were determined on the basis of similar previous studies.

### Data availability

RNA-seq data supporting the results of current study have been deposited in GEO database (https://www.ncbi.nlm.nih.gov/geo, with accession code GSE107516). Sanger sequencing data of this study have been deposited in figshare (https://figshare.com, with the identifier 10.6084/m9.figshare.6015881). Public datasets are available from GEO database (https://www.ncbi.nlm.nih.gov/geo, with accession codes GSE14340, GSE16476, GSE62564, GSE10846, GSE21653, GSE24551, GSE17679, GSE37418, GSE19234, GSE42352, GSE28735, and GSE21032) or The Cancer Genome Atlas (TCGA) database (https://cancergenome.nih.gov, with accession codes TCGA-LAML, TCGA-LIHC, TCGA-HNSC, TCGA-LUAD, and TCGA-KIRC). The copy number and genetic variation data are available from Oncogenomics database (https://pob.abcc.ncifcrf.gov/cgi-bin/JK, with the identifier CHR0600P035815896), cBioPortal for Cancer Genomics (http://cbioportal.org, with search term “ARMC12”), TARGET database (https://ocg.cancer.gov/programs/target/projects/neuroblastoma), and European Genome-phenome Archive (https://www.ebi.ac.uk/ega, with accession code EGAD00001000282). All remaining data are presented within the article and Supplementary Information Files, and available from the corresponding author upon request.

## Electronic supplementary material

Supplementary Information
