# Supplementary material for: Armadillo repeat containing 12 promotes neuroblastoma progression through interaction with retinoblastoma binding protein 4
Source: Nat Commun. 2018 Jul 19;9:2829. doi: 10.1038/s41467-018-05286-2 (PMC6053364; doi:10.1038/s41467-018-05286-2)
Supplement: Supplementary file 1 — Supplementary Information [file 41467_2018_5286_MOESM1_ESM.pdf]

Supplementary Information

Armadillo repeat containing 12 promotes neuroblastoma progression through interaction with retinoblastoma binding protein 4

Li et al.

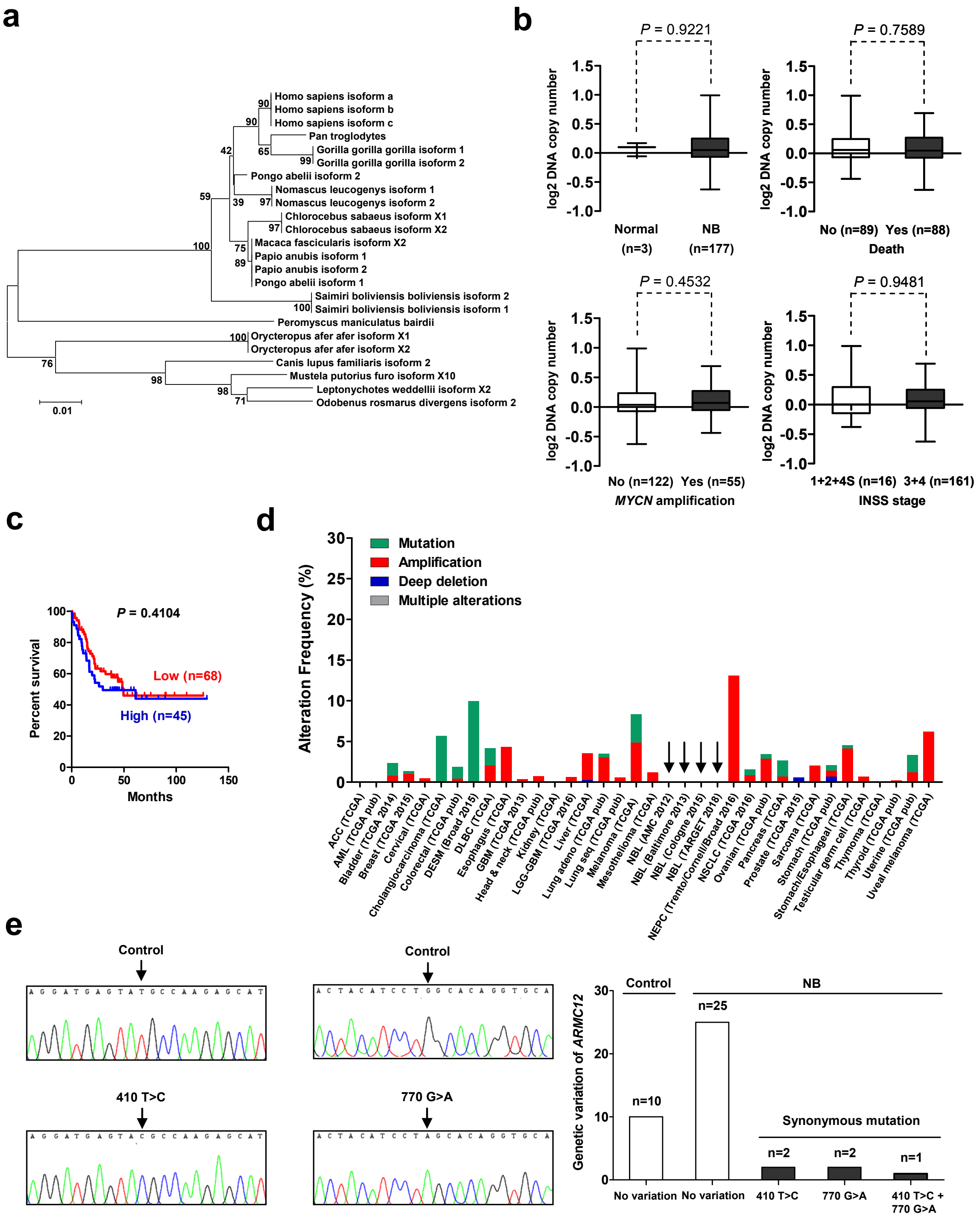

Supplementary Figure 1. Copy number and genetic variants of *ARMC12* gene in human cancers. a Phylogenetic and homology tree analyses indicating the genetic relationship of *ARMC12* in human and primate species. **b** Mining of Oncogenomics database (https://pob.abcc.ncifcrf.gov/cgi-bin/JK) revealing the copy number of *ARMC12* gene locus, locating at chr6: 35704859-35716685, in normal genomic DNA ($n = 3$) and NB tissues ($n = 177$) with varied status of death, *MYCN* amplification, or INSS stages. **c** Kaplan–Meier survival curves of 113 NB patients with high or low *ARMC12* copy number (cutoff value=0.146). **d** Mining of publicly available datasets derived from cBioPortal for Cancer Genomics (http://cbioportal.org) and European Genome-phenome Archive (EGAD00001000282) indicating the copy number alteration (n=127) and genetic variants (n=44) of *ARMC12* in common human cancers (total n = 8352), and none of these changes in 563 NB cases of three independent genome-wide studies and TARGET database (arrowheads). **e** Sanger sequencing of PCR products amplified from genomic DNA indicating two synonymous mutations within coding exons of *ARMC12* in 30 NB tissues, when compared with control peripheral blood samples from healthy age-matched pediatric population ($n = 10$). Unpaired two-sided *t*-test in **b**; log-rank test in **c**. Bars are means and whiskers (min to max) in **b**.

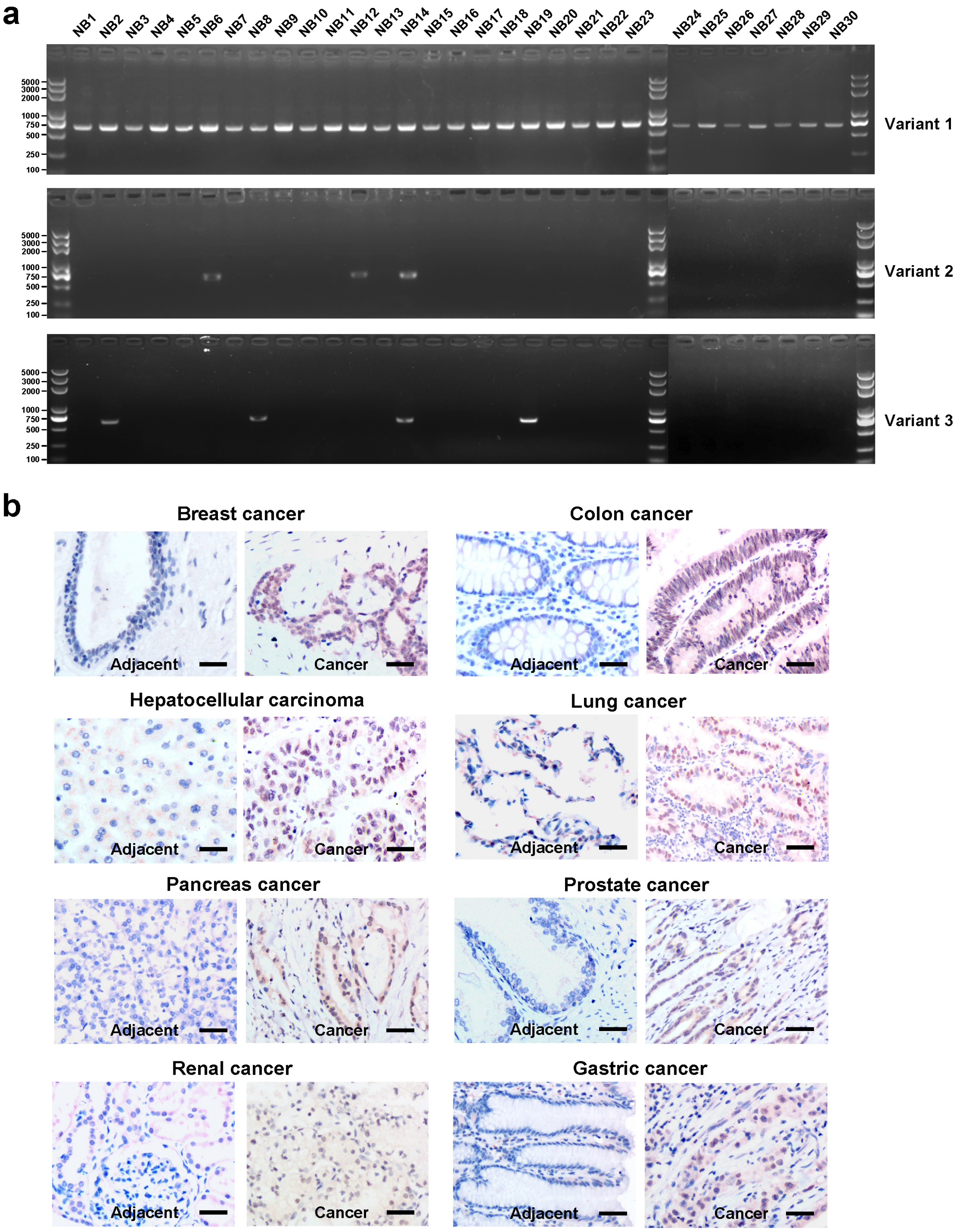

Supplementary Figure 2. Expression profiles of *ARMC12* in human cancers. a RT-PCR with specific primers showing the expression of *ARMC12* transcript variants in NB tissues ($n = 30$). **b** Representative immunohistochemical staining showing the nuclear expression of ARMC12 (brown) in the specimens of breast cancer, colon cancer, hepatocellular carcinoma, lung cancer, pancreas cancer, prostate cancer, renal cancer, and gastric cancer, but not in their adjacent normal counterparts. Scale bars: 100 μm. Data are representative of three independent experiments.

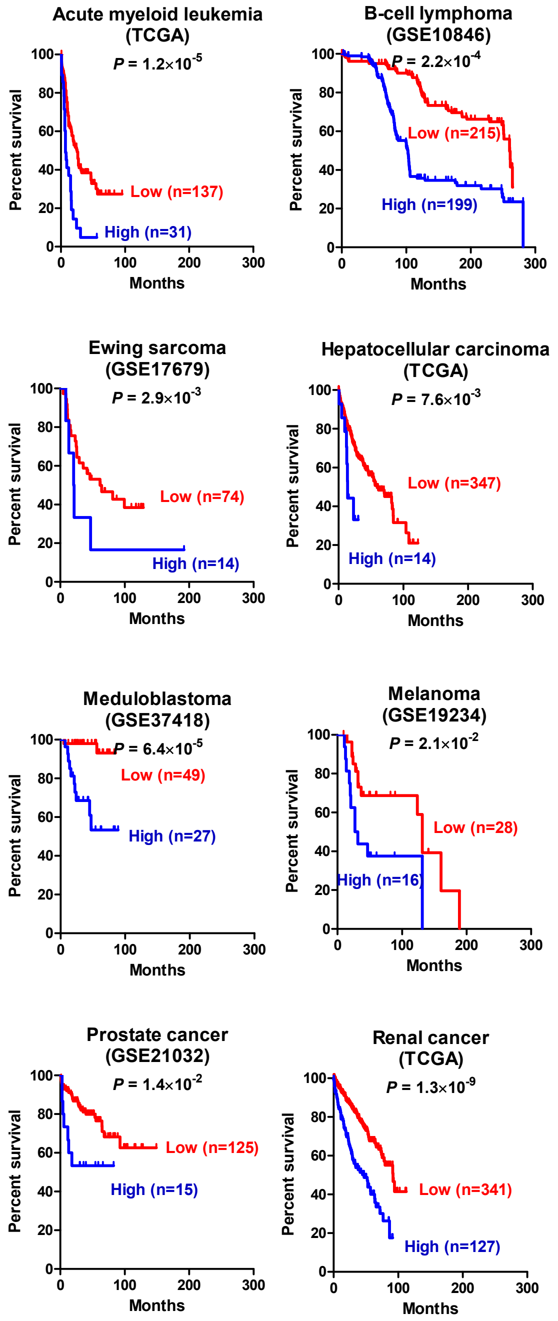

Supplementary Figure 3. Kaplan–Meier survival curves of *ARMC12* in human cancers. Mining of publicly available datasets derived from Gene Expression Omnibus (GEO) and The Cancer Genome Atlas (TCGA) indicating the survival curves of cancer patients with high or low *ARMC12* levels. Log-rank test for survival comparison.

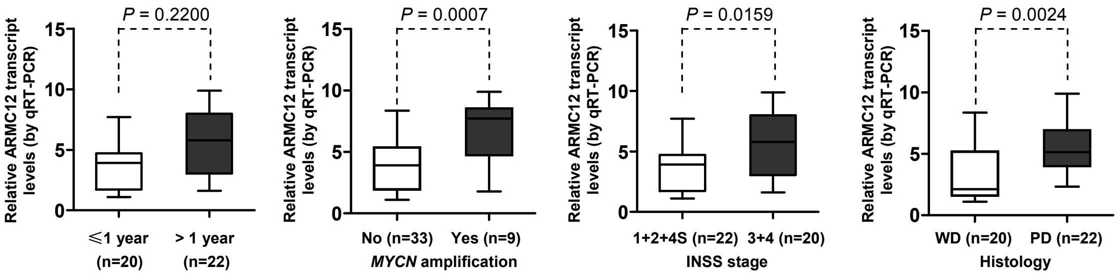

Supplementary Figure 4. Expression of *ARMC12* in 42 NB specimens. Real-time qRT-PCR indicating the *ARMC12* transcript levels (normalized to GAPDH) in 42 NB tissues with different status of patients' age, *MYCN* amplification, INSS stages, or histological differentiation. WD, well differentiation; PD, poor differentiation. Unpaired two-sided *t*-test in comparing gene expression levels. Bars are means and whiskers (min to max). Data are representative of three independent experiments.

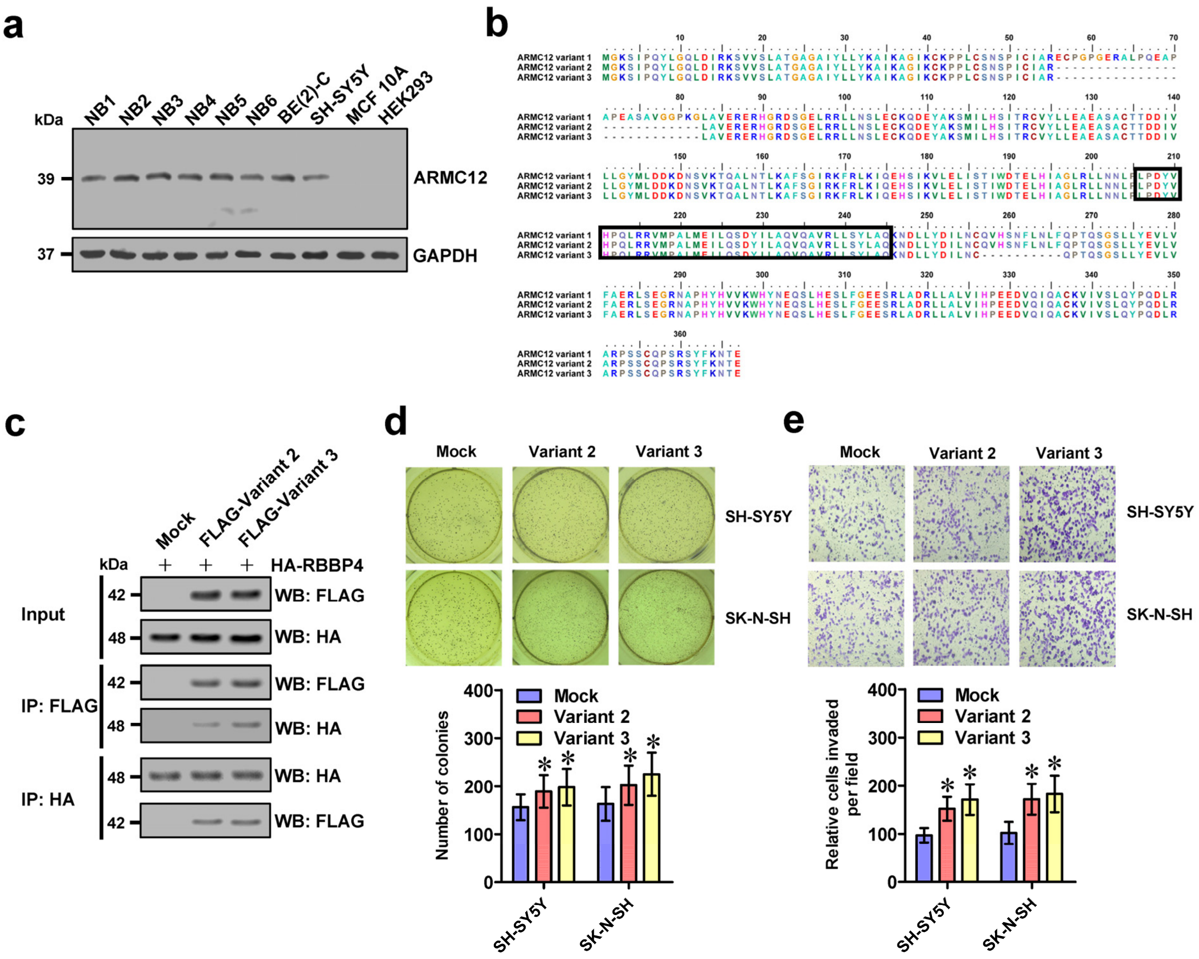

Supplementary Figure 5. Roles of *ARMC12* variants in NB cells. a Western blot assay indicating the endogenous expression of ARMC12 in NB tissues, NB cell lines, MCF 10A cells, and HEK293 cells. **b** Schematic diagram showing the amino acid similarity, especially the outlined ARM2 domain, of *ARMC12* variants. **c** Co-IP and western blot assays depicting the interaction between ARMC12 and RBBP4 protein in SH-SY5Y cells transfected with empty vector (mock), FLAG-tagged *ARMC12* variants, and HA-tagged *RBBP4*. **d**, **e** Representative images (upper panel) and quantification (lower panel) of soft agar (d) and matrigel invasion (e) assays showing the anchor-independent growth and invasion capability of NB cells stably transfected with mock or *ARMC12* variant ($n = 4$ per group). $^*P<0.01$ vs. mock (unpaired two-sided t-test in **d** and **e**). Data are shown as mean ± s.e.m. (error bars) and representative of three independent experiments.

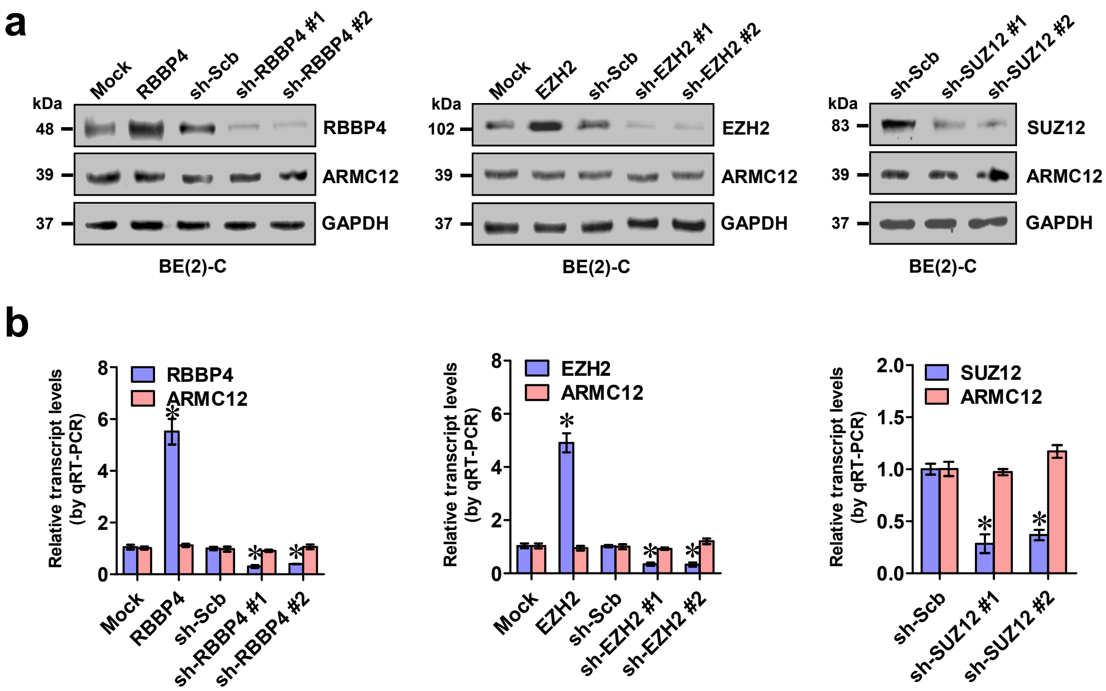

Supplementary Figure 6. Expression of *ARMC12*, *RBBP4*, *EZH2*, and *SUZ12* in NB cells. a Western blot showing the ARMC12, RBBP4, EZH2, and SUZ12 protein levels in BE(2)-C cells stably transfected with empty vector (mock), *RBBP4*, *EZH2*, scramble shRNA (sh-Scb), sh-RBBP4, sh-EZH2, or sh-SUZ12. **b** Real-time qRT-PCR indicating the transcript levels of *ARMC12*, *RBBP4*, *EZH2*, and *SUZ12* (normalized to GAPDH) in BE(2)-C cells stably transfected with mock, *RBBP4*, *EZH2*, sh-Scb, sh-RBBP4, sh-EZH2, or sh-SUZ12 ($n=5$ per group). *$P<0.01$ vs. mock or sh-Scb (unpaired two-sided t-test in **b**). Data are shown as mean ± s.e.m. (error bars) and representative of three independent experiments.

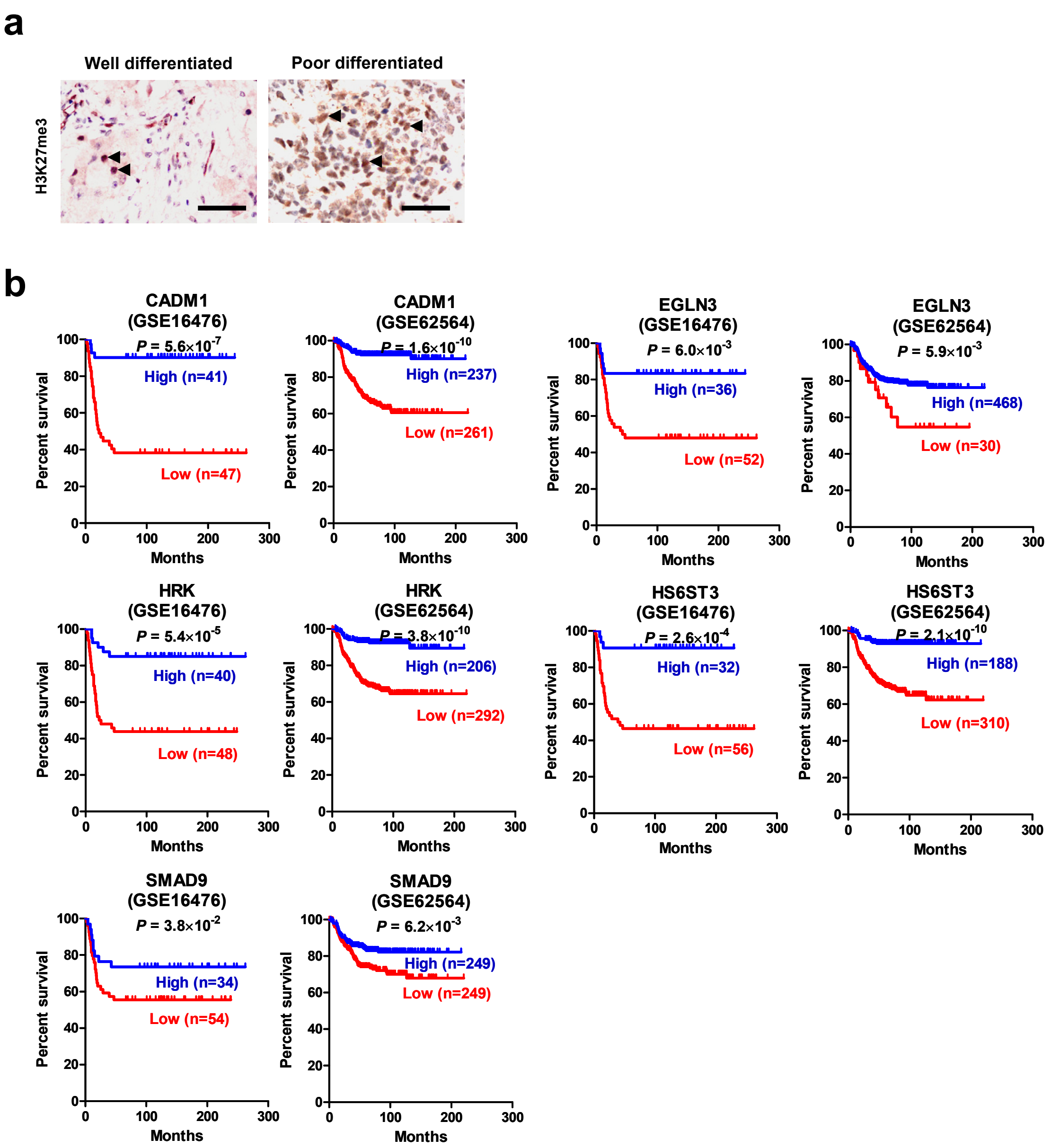

Supplementary Figure 7. Expression of H3K27me3 and Kaplan–Meier survival curves of *ARMC12* downstream genes. **a** Representative immunohistochemical images showing the expression of H3K27me3 in the tumor cells of NB specimens (arrowheads, brown). Scale bars: 100 µm. **b** Mining of publicly available microarray (GSE16476) and RNA-seq (GSE62564) datasets indicating the survival curve of NB patients with high or low levels of *CADM1* (cutoff values=2333.8 and 275.1), *EGLN3* (cutoff values=90.2 and 5.2), *HRK* (cutoff values=179.7 and 4.4), *HS6ST3* (cutoff values=53.8 and 24.2), or *SMAD9* (cutoff values=608.1 and 39.3). Log-rank test in **b**.

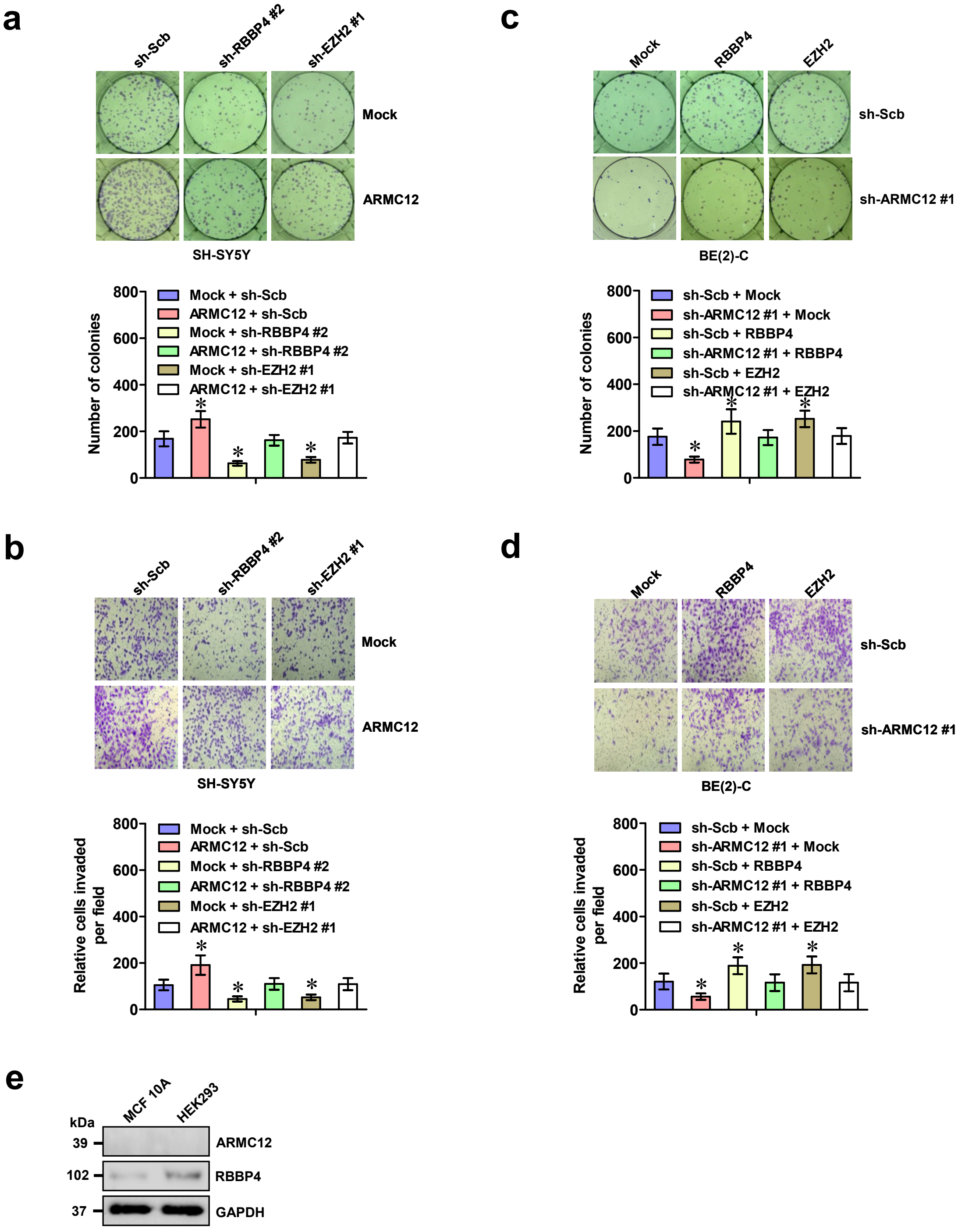

Supplementary Figure 8. Effects of *ARMC12*, *RBBP4*, and *EZH2* on NB cells. a, **c** Representative images (upper panel) and quantification (lower panel) of colony formation assay showing the growth of NB cells stably transfected with empty vector (mock), *ARMC12*, *RBBP4*, *EZH2*, scramble shRNA (sh-Scb), sh-RBBP4 #2, sh-EZH2 #1, or sh-ARMC12 #1 (n=4 per group). **b**, **d** Representative images (upper panel) and quantification (lower panel) of matrigel invasion assay indicating the invasion capability of NB cells stably transfected with mock, *ARMC12*, *RBBP4*, *EZH2*, sh-Scb, sh-RBBP4 #2, sh-EZH2 #1, or sh-ARMC12 #1 ($n = 4$ per group). **e** Western blot showing the expression levels of ARMC12 and RBBP4 (normalized to GAPDH) in MCF 10A and HEK293 cells. *P<0.01 vs. mock+sh-Scb (unpaired two-sided t-test in **a**-**d**). Data are shown as mean ± s.e.m. (error bars) and representative of three independent experiments.

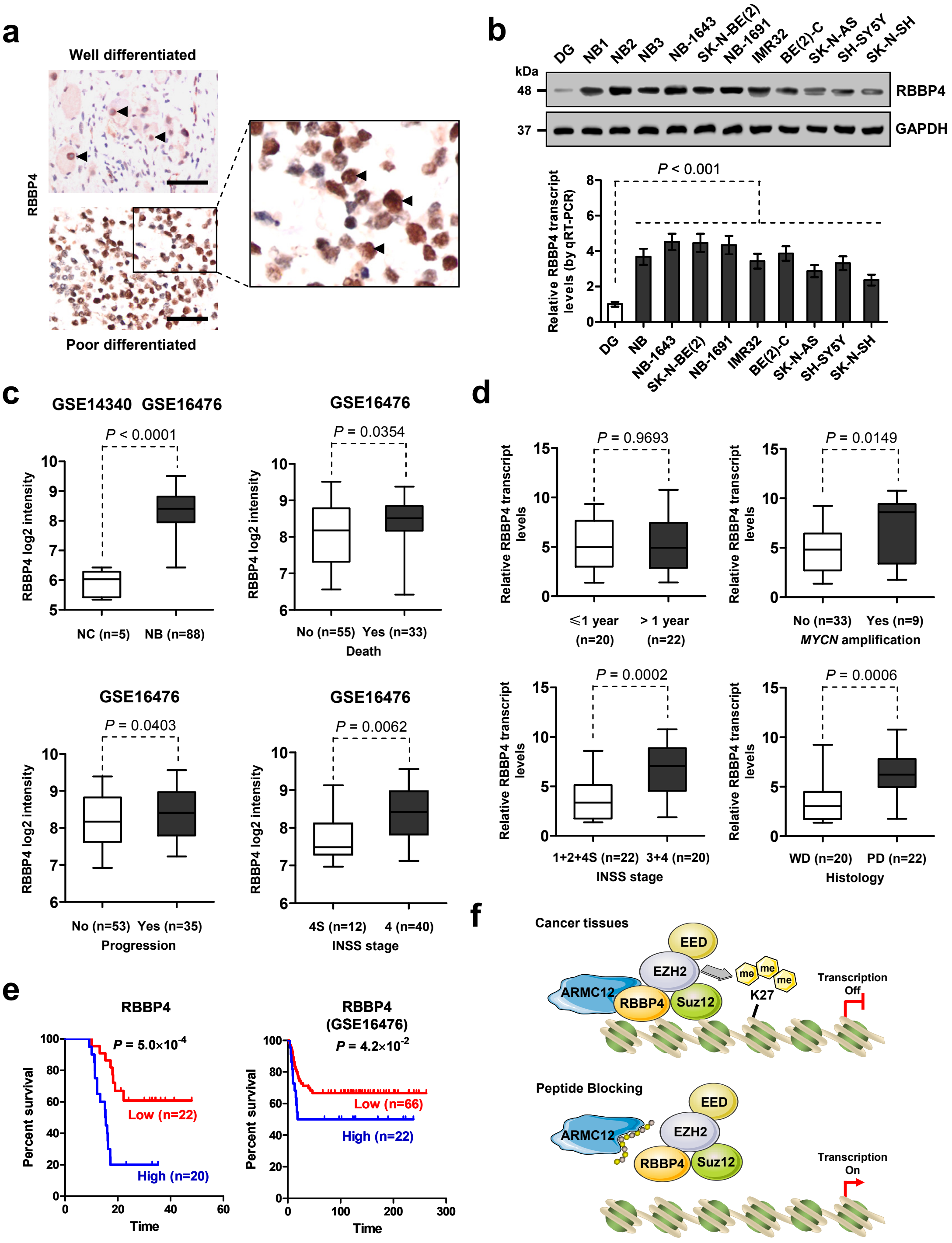

Supplementary Figure 9. High *RBBP4* expression is associated with poor outcome of NB. a Representative immunohistochemical images showing RBBP4 expression in tumor cells of NB specimens (arrowheads, brown). Scale bars: 100 μm. **b** Western blot (upper panel) and real-time qRT-PCR (lower panel) indicating the *RBBP4* levels (normalized to GAPDH) in normal dorsal ganglia (DG, n=21), NB tissues (n=42) and cell lines. **c** Mining of public datasets (GSE14340 and GSE16476) revealing the *RBBP4* transcript levels in neural crest (NC, n=5) and NB tissues (n=88). **d** Real-time qRT-PCR indicating the *RBBP4* transcript levels (normalized to GAPDH) in 42 NB tissues with different status of patients' age, *MYCN* amplification, INSS stages, or histological differentiation. WD, well differentiation; PD, poor differentiation. **e** Kaplan–Meier survival curve of 42 NB patients with high or low RBBP4 immunostaining, and that of 88 NB cases (GSE16476, cutoff value=681.9) with high or low *RBBP4* transcript levels. **f** The mechanisms underlying *ARMC12*-promoted NB progression: as a member of ARM family protein, ARMC12 interacts with RBBP4 to facilitate the PRC2 formation and EZH2 activity, resulting in transcriptional repression of downstream tumor suppressive genes associated with NB progression. One-way ANOVA with Bonferroni's multiple comparison test in **b**; unpaired two-sided t-test in **c** and **d**; log-rank test in **e**. Bars are means and whiskers (min to max) in **c** and **d**. Data are shown as mean ± s.e.m. (error bars) and representative of three independent experiments in **b**.

Figure 1f

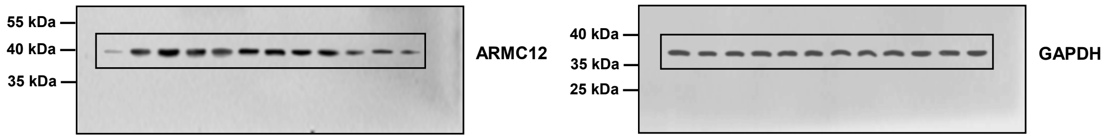

Figure 2a

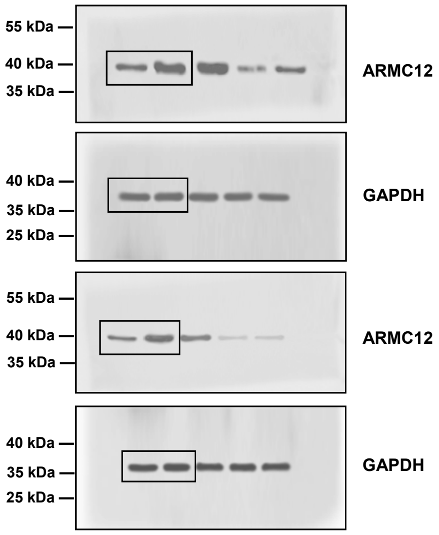

Figure 2b

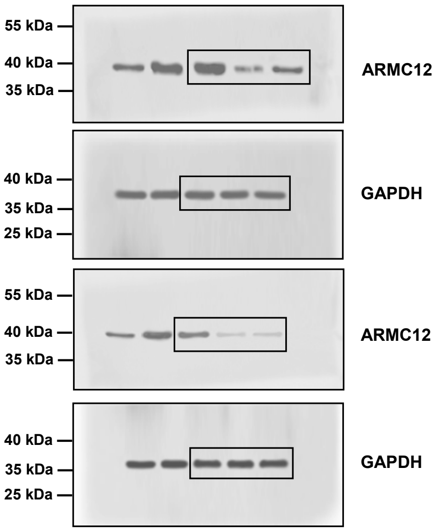

Figure 3c

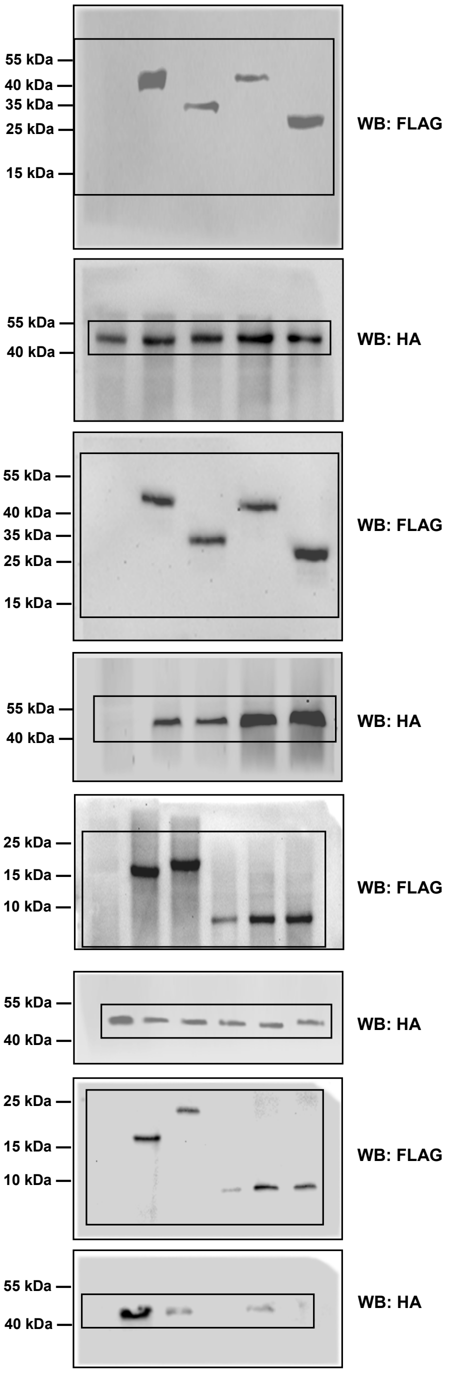

Figure 3b

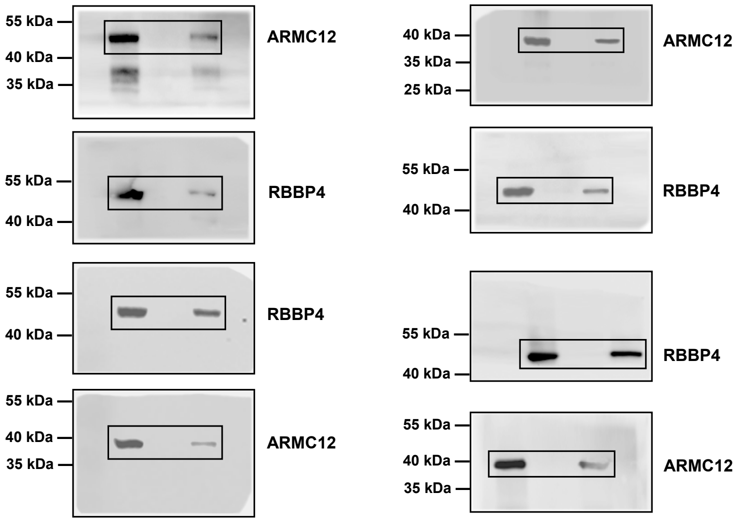

Figure 3d

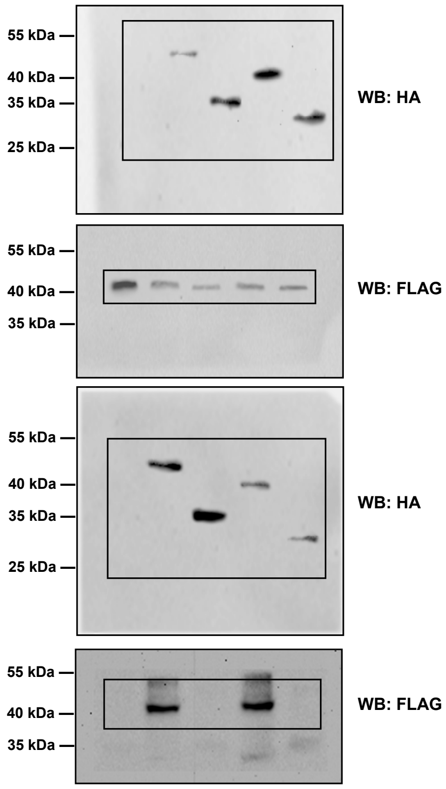

Figure 3e

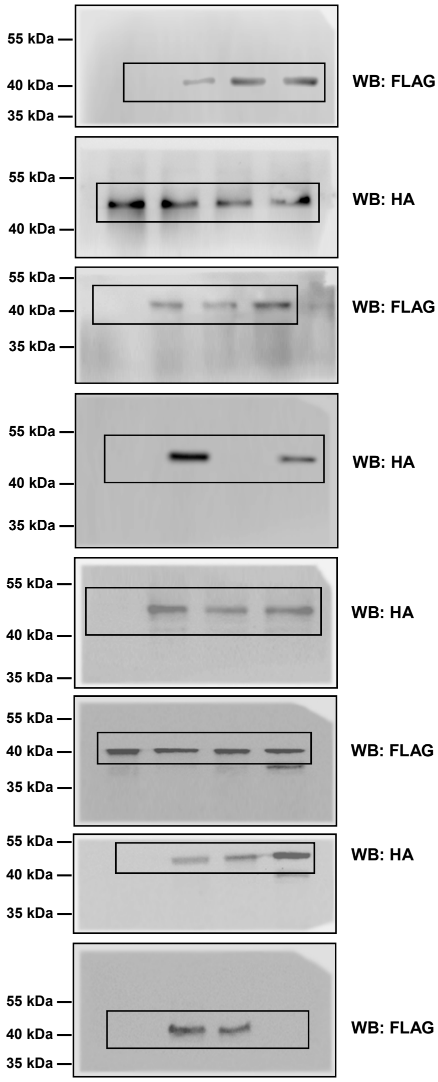

Figure 4a

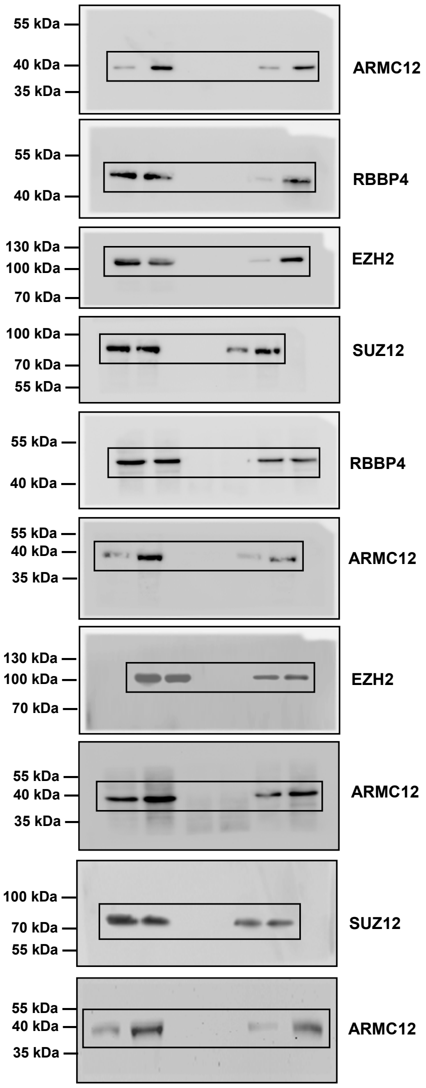

Figure 4b

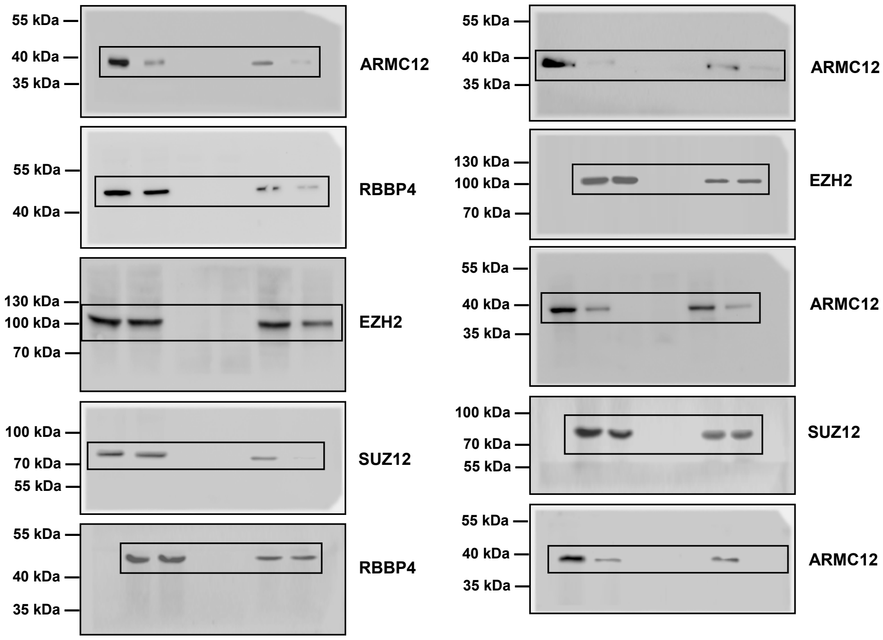

Figure 4c

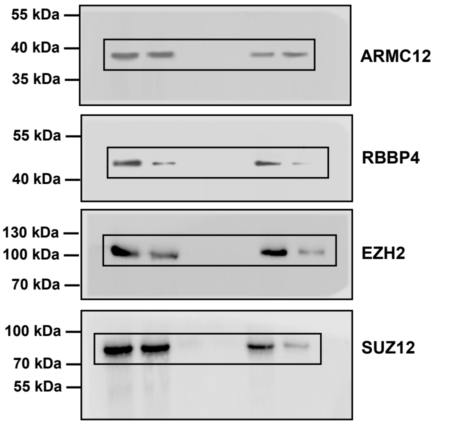

Figure 4d

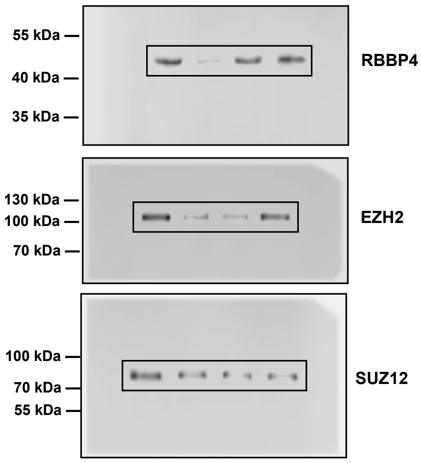

Figure 4f

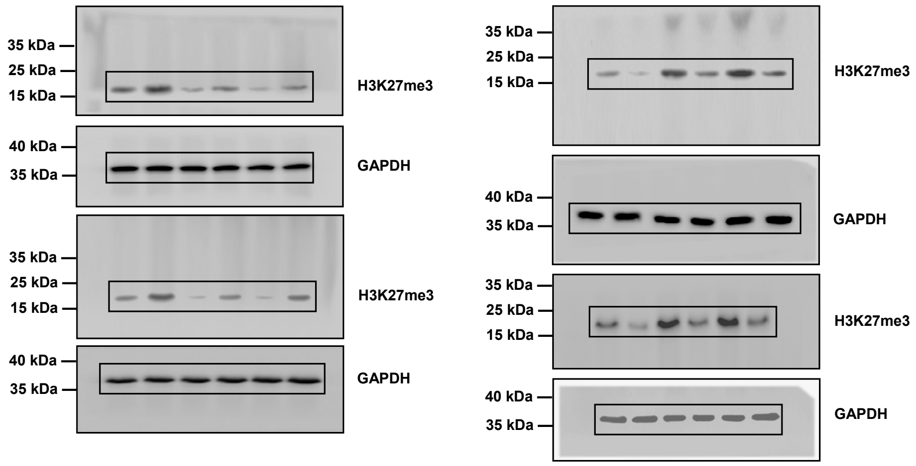

Figure 5d

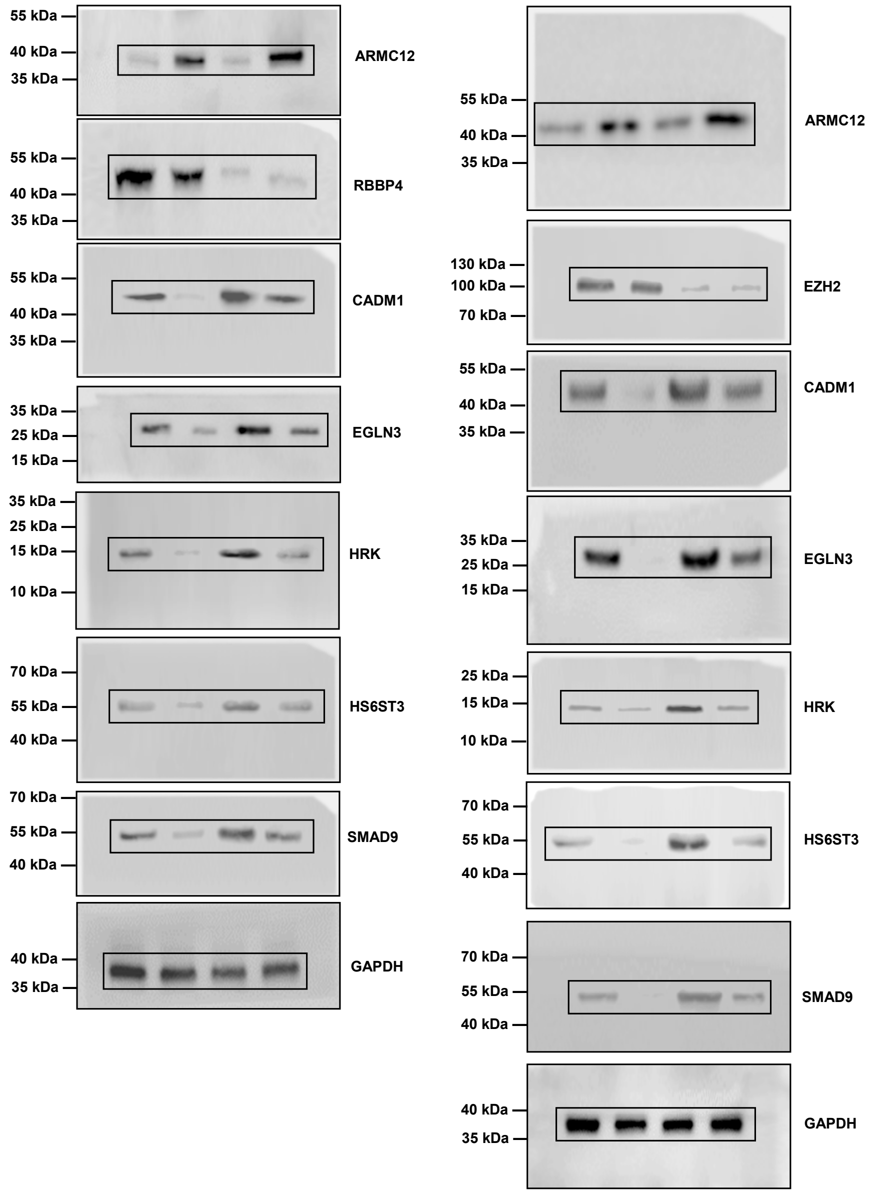

Figure 6c

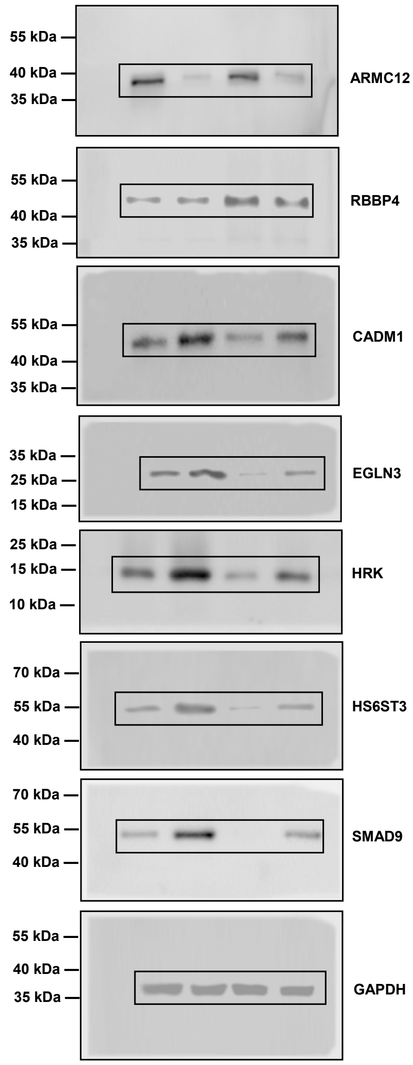

Figure 6c (continued)

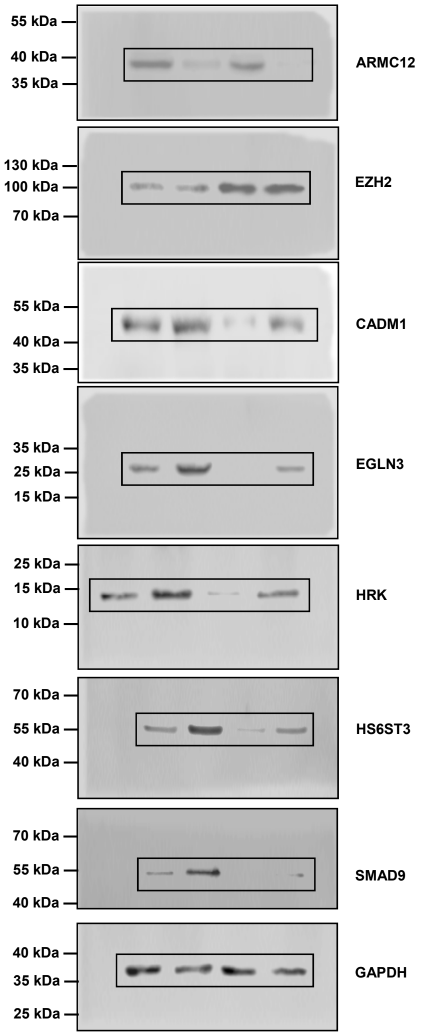

Figure 7d

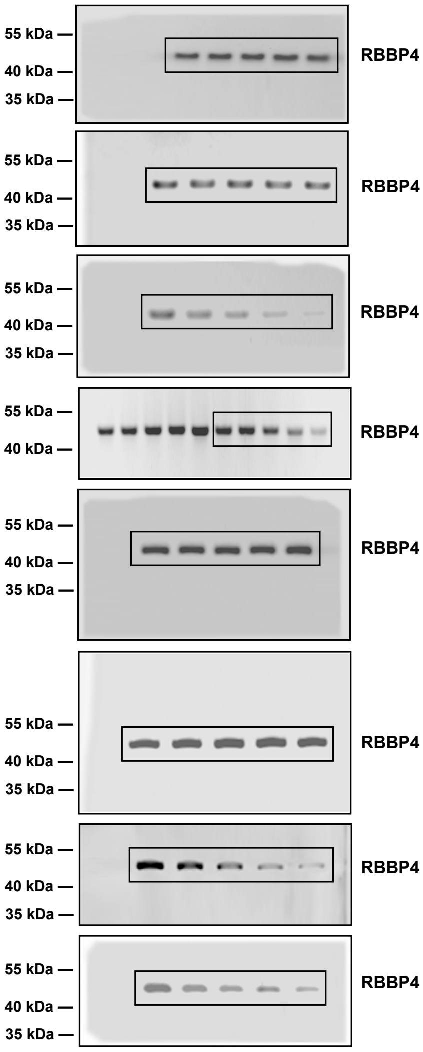

Figure 7g

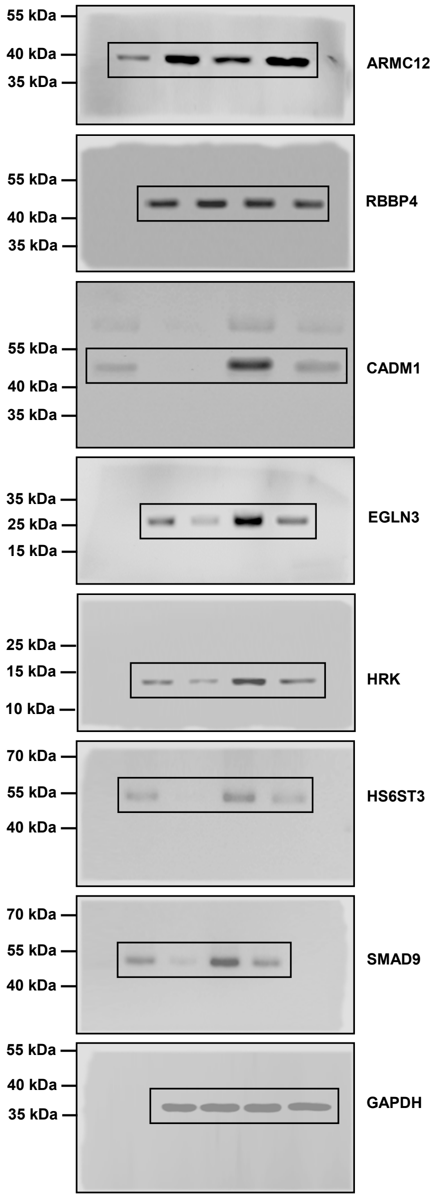

Figure 7c

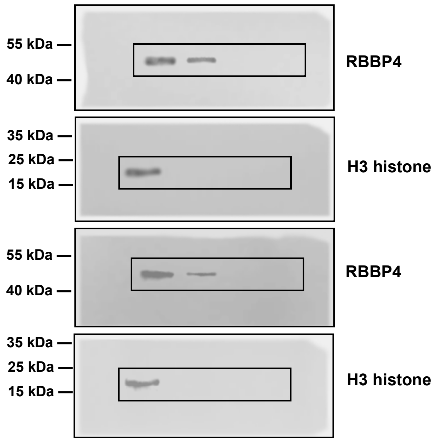

Figure 7g (continued)

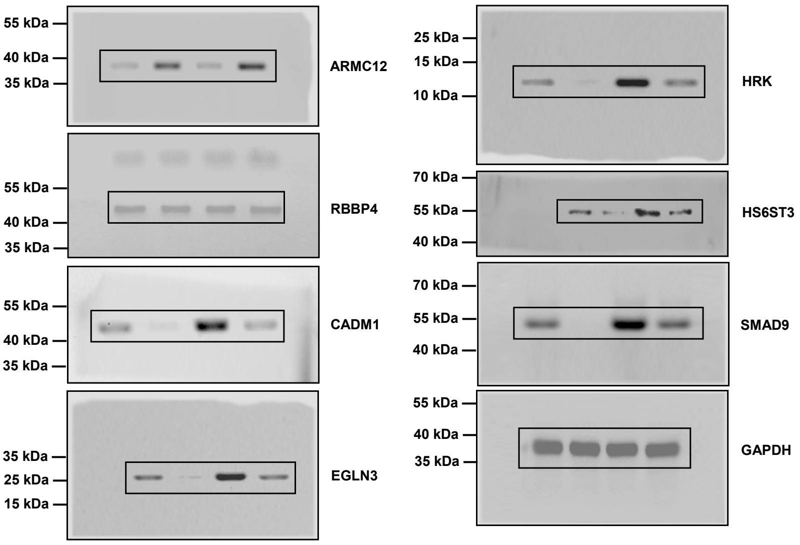

Figure 8d

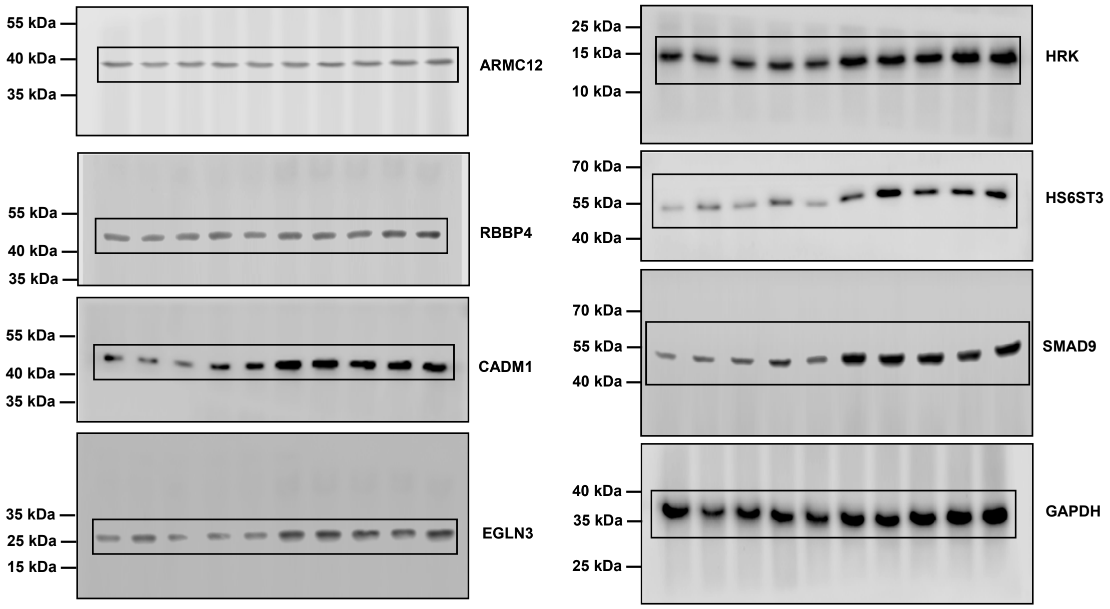

Supplementary Figure 10. Uncropped scans of western blots included in main figures.

Supplementary Table 1 ARMC12 levels in neural crest (GSE14340) and 88 NB patients (GSE16476)

NC (GSE14340)		NB (GSE16476)				
Case	ARMC12 levels	Case	Death	Progression	INSS stage	ARMC12 levels
1	2.58	1	No	No	2	4.29
2	0.00	2	No	No	2	4.60
3	4.19	3	No	Yes	4S	4.92
4	4.45	4	No	No	2	4.81
5	3.22	5	No	Yes	2	5.41
		6	No	No	4	3.90
		7	Yes	Yes	4	5.00
		8	Yes	Yes	4	4.96
		9	Yes	Yes	4	7.16
		10	Yes	Yes	4	5.96
		11	Yes	Yes	4	4.44
		12	Yes	Yes	4	4.24
		13	Yes	Yes	4	4.23
		14	Yes	Yes	4	5.43
		15	Yes	Yes	4	5.77
		16	No	No	4	4.73
		17	Yes	No	3	4.51
		18	No	No	3	4.97
		19	Yes	Yes	4	4.57
		20	No	No	4	4.96
		21	No	No	2	5.38
		22	No	No	4S	3.92
		23	Yes	Yes	4	6.00
		24	No	No	4S	4.14
		25	No	No	3	3.84
		26	No	Yes	4S	3.29
		27	No	No	2	4.31
		28	No	No	3	4.54
		29	No	No	4S	4.36
		30	No	No	3	5.07
		31	No	No	1	4.91
		32	Yes	Yes	4	4.77
		33	Yes	No	4	4.87
		34	No	Yes	2	4.47
		35	No	No	4	4.53
		36	No	No	1	4.76
		37	Yes	Yes	4	5.22
		38	No	Yes	4S	4.35
		39	No	No	2	5.13
		40	No	No	1	4.73
		41	Yes	Yes	4	5.10
		42	No	No	3	4.43
		43	No	No	4S	2.58
		44	No	No	3	5.57
		45	Yes	Yes	4	5.12
		46	No	No	4	2.85
		47	Yes	Yes	4	5.02
		48	Yes	Yes	2	2.29
		49	No	No	4S	4.99
		50	No	No	2	4.67
		51	No	No	1	4.01
		52	No	No	2	5.04
		53	No	No	2	5.12
		54	No	No	4	6.13
		55	No	No	3	4.78
		56	No	No	4S	5.02
		57	No	No	3	4.53
		58	Yes	No	4	5.89
		59	No	No	4S	4.69
		60	No	No	4	4.05
		61	Yes	Yes	4	4.86
		62	No	No	4	4.51
		63	No	No	1	5.01
		64	No	No	3	4.50
		65	Yes	Yes	4	5.97
		66	Yes	Yes	4	4.40
		67	No	No	2	5.13
		68	No	No	1	5.01
		69	Yes	Yes	4	6.01
		70	Yes	Yes	4	5.49
		71	No	No	4	4.26
		72	No	No	1	4.21
		73	Yes	Yes	4	4.87
		74	No	No	4	4.11
		75	Yes	Yes	4	5.03
		76	No	No	2	5.22
		77	Yes	Yes	3	4.02
		78	No	No	1	4.39
		79	No	No	4S	4.63
		80	No	No	3	5.68
		81	Yes	Yes	4	5.42
		82	Yes	Yes	4	5.21
		83	No	No	2	5.40
		84	Yes	Yes	4	4.88
		85	Yes	Yes	4	4.96
		86	No	No	4	4.48
		87	Yes	Yes	3	5.63
		88	No	No	4S	3.58

NC, neural crest; INSS, international neuroblastoma staging system.

Supplementary Table 2 Genetic and transcript variants of *ARMC12* in 30 NB cases

Case	Age (months)	Gender	*MYCN* amplification	INSS stage	Histology	Synonymous genetic variant	Transcript variant
1	6.2	F	No	4	PD	N	1
2	5.3	F	No	4S	PD	N	1, 3
3	17.1	M	No	2	PD	N	1
4	10.2	M	No	4S	PD	N	1
5	16.3	F	No	4S	PD	N	1
6	8.1	F	No	4S	PD	N	1, 2
7	9.2	M	No	2	PD	N	1
8	33.5	F	No	4S	PD	410 T>C, 770 G>A	1, 3
9	22.4	M	No	4	PD	N	1
10	58.2	M	Yes	4S	PD	N	1
11	8.1	M	Yes	3	PD	N	1
12	9	M	No	2	WD	N	1, 2
13	18.3	M	No	2	WD	770 G>A	1
14	35.1	F	No	1	WD	N	1, 2, 3
15	23.3	M	No	2	WD	N	1
16	18.2	F	No	1	WD	N	1
17	10.4	M	No	3	WD	N	1
18	16.5	M	No	3	WD	N	1
19	8.2	M	No	2	WD	N	1, 3
20	7.3	M	No	2	WD	N	1
21	11.3	F	Yes	4S	PD	770 G>A	1
22	7.4	M	No	4S	PD	N	1
23	5.2	M	Yes	4	PD	N	1
24	6.1	M	No	4	PD	410 T>C	1
25	9.3	M	No	3	PD	N	1
26	8.2	F	No	2	WD	N	1
27	7.2	M	Yes	1	WD	N	1
28	7.1	M	No	1	WD	N	1
29	10.2	M	Yes	3	WD	410 T>C	1
30	6.1	M	No	2	WD	N	1

F, female; M, male; INSS, international neuroblastoma staging system; PD, poor differentiation; WD, well differentiation; N, normal.

Supplementary Table 3 ARMC12 expression in 42 NB tissues

Group	Total number	ARMC12 expression				Positive rates (%)	*P*-Value
		−	+	++	+++		
Age							
≤1 year	20	4	6	5	5	80.0	0.922
>1 year	22	6	5	5	6	72.7	
Differentiation							
Well differentiated	20	9	4	2	5	55.0	0.013
Poorly differentiated	22	1	7	8	6	95.5	
MKI							
<200	19	9	5	3	2	52.6	0.006
>200	23	1	6	7	9	95.7	
INSS stages							
Stage 1-2	14	8	3	3	0	42.9	
Stage 3-4	20	1	7	4	8	95.0	0.008
Stage 4S	8	1	1	3	3	87.5	
MYCN amplification							
No	33	7	10	8	8	78.8	0.644
Yes	9	3	1	2	3	66.7	

ARMC12, armadillo repeat containing 12; MKI, mitosis karyorrhexis index; INSS, international neuroblastoma staging system.

Supplementary Table 4 Mass spectrometry analysis of proteins pulled down by ARMC12 antibody

SH-SY5Y			SK-N-SH		
**Mock**	**ARMC12**	**Differential protein**	**Mock**	**ARMC12**	**Differential protein**
ACTA1	ACTA1	ANXA2P2	ACTN2	ACTN2	ANXA2
ACTBL2	ACTBL2	APOC3	ACTN3	ACTN3	ASS1
ACTG1	ACTG1	ATP5B	ACTN4	ACTN4	BASP1
ANXA2	ANXA2	CALML5	ANXA2	ARMC12	DBN1
ARMC12	ANXA2P2	CORO1C	ARMC12	ASS1	EEF1A1
C3	APOC3	CORO6	ATP5A1	ATP5A1	EEF1A1P5
CHTOP	ARMC12	DDX21	ATP5B	ATP5B	EIF4A1
DDX17	ATP5B	DDX39A	EEF1A1	BASP1	FLG2
DDX21	C3	DSG1	EEF1A1P5	DBN1	FLNB
DDX5	CALML5	DSP	EIF4A1	EZH2	FSCN1
DSP	CHTOP	EEF1A2	EZH2	FLG2	H2AFV
EEF1A1	CORO1C	EZH2	FLNB	FSCN1	HIST1H2AH
EEF1A1P5	CORO6	FABP5	H2AFX	H2AFV	HIST1H4A
ELAVL1	DDX17	FBLN1	HIST1H2AH	H2AFX	HIST2H2AC
GRN	DDX39A	FLG	HIST2H2AA3	HIST1H4A	HNRNPA1
H2AFJ	DDX5	HBA1	HNRNPA1	HIST2H2AA3	HSPA8
H2AFV	DSG1	HIST1H1B	HSPA8	HIST2H2AC	JUP
H2AFX	EEF1A1	HIST1H2BM	HSPA9	HSPA9	MYH10
H2AFZ	EEF1A1P5	HNRNPA2B1	KRT1	JUP	MYH9
HIST1H1E	EEF1A2	HNRNPA3	KRT10	KRT1	PKM
HIST1H2AG	ELAVL1	HNRNPAB	PKM	KRT10	PLEC
HIST1H2AH	EZH2	HNRNPU	RPL3	MYH10	RBBP4
HIST1H2AJ	FABP5	HSPA5	SBSN	MYH9	SFPQ
HIST1H2BH	FBLN1	HSPA9	SLC2A1	PLEC	SLC2A1
HIST1H2BL	FLG	KRT1	TUBA1C	RBBP4	SUZ12
HNRNPA2B1	GRN	KRT9	TUBB	RPL3	TMOD3
HNRNPAB	H2AFJ	RBBP4		SBSN	TRIM21
HRNR	H2AFV	SEPT7		SFPQ	TUBB4B
HSPA8	H2AFX	SERPINB12		SUZ12	TUFM
HSPA9	H2AFZ	TUBA1C		TMOD3	VIM
KRT1	HBA1			TRIM21	
KRT17	HIST1H1B			TUBA1C	
RBBP7	HIST1H1E			TUBB	
RBMX	HIST1H2AG			TUBB4B	
SEPT2	HIST1H2AH			TUFM	
SUZ12	HIST1H2AJ			VIM	
	HIST1H2BH				
	HIST1H2BL				
	HIST1H2BM				
	HNRNPA3				
	HNRNPU				
	HRNR				
	HSPA5				
	HSPA8				
	KRT17				
	KRT9				
	RBBP4				
	RBBP7				
	RBMX				
	SEPT2				
	SEPT7				
	SERPINB12				
	SUZ12				
	TUBA1C				

Supplementary Table 5 Correlation between the expression of ARMC12 and H3K27me3

	ARMC12 expression			
	Low	High	*R*-value	*P*-value
**H3K27me3 expression**				
Low	17	6	0.526	<0.001
High	4	15		

ARMC12, armadillo repeat containing 12; H3K27me3, histone H3 lysine 27 trimethylation; Pearson's correlation coefficient was applied to determine the expression correlation.

Supplementary Table 6 RBBP4 expression in 42 NB tissues

Group	Total number	RBBP4 expression				Positive rates (%)	*P*-Value
		−	+	++	+++		
Age							
≤1 year	20	5	5	5	5	75.0	0.932
>1 year	22	7	5	4	6	68.2	
Differentiation							
Well differentiated	20	10	4	1	5	50.0	0.011
Poorly differentiated	22	2	6	8	6	90.9	
MKI							
<200	19	8	7	1	3	57.9	0.016
>200	23	4	3	8	8	82.6	
INSS stages							
Stage 1-2	14	7	7	0	0	50.0	
Stage 3-4	20	4	3	4	9	80.0	<0.001
Stage 4S	8	1	0	5	2	87.5	
MYCN amplification							
No	33	11	8	7	7	66.7	0.441
Yes	9	1	2	2	4	88.9	

RBBP4, retinoblastoma binding protein 4; MKI, mitosis karyorrhexis index; INSS, international neuroblastoma staging system.

Supplementary Table 7 Oligonucleotide sets used for constructs and short hairpin RNAs

Oligo Set	Sequences
pcDNA3.1-ARMC12	5'-CGCGGATCCATGGGCAAGAGCATCCCCCAAT-3' (sense);<br>5'-CCGCTCGAGTTATTCCGTGTTTTTAAAGTAGG-3' (antisense)
pCMV-3Tag-1A-ARMC12	5'-CGCGGATCCATGGGCAAGAGCATCCCCCAAT-3' (sense);<br>5'-GCCGCTCGAGTTATTCCGTGTTTTTAAAGTAGGAACG-3' (antisense)
pCMV-3Tag-1A-ARMC12 (△N)	5'-CGCGGATCCCTGGAGGCTGAGGCCTCTGCTT-3' (sense);<br>5'-GCCGCTCGAGTTATTCCGTGTTTTTAAAGTAGGAACG-3' (antisense)
pCMV-3Tag-1A-ARMC12 (△C)	5'-CGCGGATCCATGGGCAAGAGCATCCCCCAAT-3' (sense);<br>5'-GCCGCTCGAGATACTGCAGGCTGACAATGACC-3' (antisense)
pCMV-3Tag-1A-ARMC12 (ARM1-3)	5'-CGCGGATCCCTGGAGGCTGAGGCCTCTGCTT-3' (sense);<br>5'-GCCGCTCGAGATACTGCAGGCTGACAATGACC-3' (antisense)
pCMV-3Tag-1A-ARMC12 (ARM1-2)	5'-CGCGGATCCCTGGAGGCTGAGGCCTCTGCTT-3' (sense);<br>5'-GCCGCTCGAGCTGTGCCAGGTAGCTCAGCAGT-3' (antisense)
pCMV-3Tag-1A-ARMC12 (ARM2-3)	5'-CGCGGATCCCTGCCCGACTATGTGCATCCAC-3' (sense);<br>5'-GCCGCTCGAGATACTGCAGGCTGACAATGACC-3' (antisense)
pCMV-3Tag-1A-ARMC12 (ARM1)	5'-CGCGGATCCCTGGAGGCTGAGGCCTCTGCTT-3' (sense);<br>5'-GCCGCTCGAGGCCAGAGAAAGCTTTAAGTGTA-3' (antisense)
pCMV-3Tag-1A-ARMC12 (ARM2)	5'-CGCGGATCCCTGCCCGACTATGTGCATCCAC-3' (sense);<br>5'-GCCGCTCGAGCTGTGCCAGGTAGCTCAGCAGT-3' (antisense)
pCMV-3Tag-1A-ARMC12 (ARM3)	5'-CGCGGATCCTCCCTGCATGAATCCCTCTTTG-3' (sense);<br>5'-GCCGCTCGAGATACTGCAGGCTGACAATGACC-3' (antisense)
pCMV-3Tag-1A-ARMC12 (ARM Mut #1)	5'-CTGCGACGGGCAATGCCTGCCTTGATGGAGATCCTGCAGTCAG-3' (sense);<br>5'-AAGGCAGGCATTGCCCGTCGCAGCTGTGGATGCACATAGTCGG-3' (antisense)
pCMV-3Tag-1A-ARMC12 (ARM Mut #2)	5'-GGAGATCCTGGCGGCAGACTACATCCTGGCACAGGTGCAAGCCGT-3' (sense);<br>5'-GATGTAGTCTGCCGCCAGGATCTCCATCAAGGCAGGCATCACCCG-3' (antisense)
pCMV-3Tag-1A-ARMC12 (variant Mut #1)	5'-CATCTGCATCGCCCGCCTGGCAGTCGAGCGAGAGCGGCAC-3' (sense);<br>5'-CGCTCGACTGCCAGGCGGGCGATGCAGATGGGTGAGTTGC-3' (antisense)
pCMV-3Tag-1A-ARMC12 (variant Mut #2)	5'-ATTCTCAACTGCCAGCCCACACAGTCAGGGAGTCTCCTGT-3' (sense);<br>5'-CCCTGACTGTGTGGGCTGGCAGTTGAGAATGTCATAGAGA-3' (antisense)
pBiFC-ARMC12-VN173	5'-CGGGGTACCGATGGGCAAGAGCATCCCCCAATAC-3' (sense);<br>5'-CTAGTCTAGATTCCGTGTTTTTAAAGTAGGAACG-3' (antisense)
pBiFC-ARMC12-VN173 (ARM Mut #1)	5'-CTGCGACGGGCAATGCCTGCCTTGATGGAGATCCTGCAGTCAG-3' (sense);<br>5'-AAGGCAGGCATTGCCCGTCGCAGCTGTGGATGCACATAGTCGG-3' (antisense)
pBiFC-ARMC12-VN173 (ARM Mut #2)	5'-GGAGATCCTGGCGGCAGACTACATCCTGGCACAGGTGCAAGCCGT-3' (sense);<br>5'-GATGTAGTCTGCCGCCAGGATCTCCATCAAGGCAGGCATCACCCG-3' (antisense)
pCMV-HA-RBBP4	5'-CGGAAGATCTGGATGGCCGACAAGGAAGCAGCCTTCGA-3' (sense);<br>5'-GCCGCTCGAGCTAGGACCCTTGTCCTTCTGGATCCA-3' (antisense)
pCMV-HA-RBBP4 (△N)	5'-CGGAAGATCTGGAACCATGAAGGAGAAGTAAACAGGGC-3' (sense);<br>5'-GCCGCTCGAGCTAGGACCCTTGTCCTTCTGGATCCA-3' (antisense)
pCMV-HA-RBBP4 (△C)	5'-CGGAAGATCTGGATGGCCGACAAGGAAGCAGCCTTCGA-3' (sense);<br>5'-GCCGCTCGAGTTGCCACACTTGCATGATATTGTC-3' (antisense)
pCMV-HA-RBBP4 (WD40)	5'-CGGAAGATCTGGAACCATGAAGGAGAAGTAAACAGGGC-3' (sense);<br>5'-GCCGCTCGAGTTGCCACACTTGCATGATATTGTC-3' (antisense)
pCMV-HA-RBBP4 (Mut #1)	5'-AAAGAACACCGCTGCTGCTTATGATTTGGTGATGACCCATGCTCTGGAG-3' (sense);<br>5'-CCAAATCATAAGCAGCAGCGGTGTTCTTTTTCCATATTTTGTATTCCTC-3' (antisense)
pCMV-HA-RBBP4 (Mut #2)	5'-TATGATTTGGCGATGACCCATGCTCTGGAGTGGCCCAGCCTA-3' (sense);<br>5'-CATGGGTCATCGCCAAATCATAAAGAAAAGGGGTGTTCTTTT-3' (antisense)
pBiFC-RBBP4-VC155	5'-CGGAAGATCTGGATGGCCGACAAGGAAGCAGCCTTC-3' (sense);<br>5'-CGGGGTACCGGACCCTTGTCCTTCTGGATCCAC-3' (antisense)
pBiFC-RBBP4-VC155 (Mut #1)	5'-AAAGAACACCGCTGCTGCTTATGATTTGGTGATGACCCATGCTCTGGAG-3' (sense);<br>5'-CCAAATCATAAGCAGCAGCGGTGTTCTTTTTCCATATTTTGTATTCCTC-3' (antisense)
pBiFC-RBBP4-VC155 (Mut #2)	5'-TATGATTTGGCGATGACCCATGCTCTGGAGTGGCCCAGCCTA-3' (sense);<br>5'-CATGGGTCATCGCCAAATCATAAAGAAAAGGGGTGTTCTTTT-3' (antisense)
pCMV-N-myc-EZH2	5'-CGCGGATCCATGGGCCAGACTGGGAAGAAATCTGA-3' (sense);<br>5'-GCCGCTCGAGTCAAGGGATTTCCATTTCTCTTTCGATGC-3' (antisense)
pCMV-N-myc-SUZ12	5'-GCCGCTCGAGATGGCGCCTCAGAAGCACGGCGGTGG-3' (sense);<br>5'-CTAGTCTAGATCAGAGTTTTTGTTTTTTGCTCTGTTTTG-3' (antisense)
sh-Scb	5'-AGGGATACAAGCATATACCACTCGAGTGGTATATGCTTGTATCCCTC-3' (sense);<br>5'-GAGGGATACAAGCATATACCACTCGAGTGGTATATGCTTGTATCCCT-3' (antisense)
sh-ARMC12 #1	5'-CCACGTGGTGAAATGGCATTACTCGAGTAATGCCATTTCACCACGTGG-3' (sense);<br>5'-CCACGTGGTGAAATGGCATTACTCGAGTAATGCCATTTCACCACGTGG-3'(antisense)
sh-ARMC12 #2	5'-CCGGCACAGAAGAATGACCTTCTCTCGAGAGAAGGTCATTCTTCTGTGCC-3'(sense);<br>5'-CCGGCACAGAAGAATGACCTTCTCTCGAGAGAAGGTCATTCTTCTGTGCC-3' (antisense)
sh-RBBP4 #1	5'-CCGGGCCTTTCTTTCAATCCTTATACTCGAGTATAAGGATTGAAAGAAAGGCTTTTTG -3' (sense);<br>5'-AATTCAAAAAGCCTTTCTTTCAATCCTTATACTCGAGTATAAGGATTGAAAGAAAGGC -3' (antisense)
sh-RBBP4 #2	5'-CCGGCCCTTGTATCATCGCAACAAACTCGAGTTTGTTGCGATGATACAAGGGTTTTTG-3' (sense);<br>5'-AATTCAAAAACCCTTGTATCATCGCAACAAACTCGAGTTTGTTGCGATGATACAAGGG-3' (antisense)
sh-EZH2 #1	5'-CCGGGCTAGGTTAATTGGGACCAAACTCGAGTTTGGTCCCAATTAACCTAGCTTTTTG -3' (sense);<br>5'-AATTCAAAAAGCTAGGTTAATTGGGACCAAACTCGAGTTTGGTCCCAATTAACCTAGC-3' (antisense)
sh-EZH2 #2	5'-CCGGCCCAACATAGATGGACCAAATCTCGAGATTTGGTCCATCTATGTTGGGTTTTTG-3' (sense);<br>5'-AATTCAAAAACCCAACATAGATGGACCAAATCTCGAGATTTGGTCCATCTATGTTGGG-3' (antisense)
sh-SUZ12 #1	5'-CCGGCCAAACCTCTTGCCACTAGAACTCGAGTTCTAGTGGCAAGAGGTTTGGTTTTTG-3' (sense);<br>5'-AATTCAAAAACCAAACCTCTTGCCACTAGAACTCGAGTTCTAGTGGCAAGAGGTTTGG-3' (antisense)
sh-SUZ12 #2	5'-CCGGGCTTACGTTTACTGGTTTCTTCTCGAGAAGAAACCAGTAAACGTAAGCTTTTTG-3' (sense);<br>5'-AATTCAAAAAGCTTACGTTTACTGGTTTCTTCTCGAGAAGAAACCAGTAAACGTAAGC-3' (antisense)

Supplementary Table 8 Primer sets used for PCR and ChIP

Primer set	Primers	Sequence	Product size (bp)	Application
ARMC12	Forward	5'-CAAAAGCGTAGTCAGCCTGG-3'	115	qPCR
	Reverse	5'-GATGCAGATGGGTGAGTTGC-3'		
ARMC12	Forward	5'-GCATCGCCCGTGAGTGTC-3'	638	RT-PCR
(variant 1)	Reverse	5'-GTTTAGGAAGTTGGAGTGA-3'		
ARMC12	Forward	5'-CATCGCCCGCCTGGCAGT-3'	556	RT-PCR
(variant 2)	Reverse	5'-GTTTAGGAAGTTGGAGTGA-3'		
ARMC12	Forward	5'-CATCGCCCGCCTGGCAGT-3'	544	RT-PCR
(variant 3)	Reverse	5'-CTGTGTGGGCTGGCAGTT-3'		
ARMC12	Forward	5'-AGAGTTCTGGTTCCGGAAGG-3'	321	PCR
(exon 1)	Reverse	5'-CTTTGGGCCCTCCCACAGCA-3'		
ARMC12	Forward	5'-GCCTGGCAGTCGAGCGAGAG-3'	146	PCR
(exon 2)	Reverse	5'-CTCAGCCTCCAGCAAGTACA-3'		
ARMC12	Forward	5'-GCCTCTGCTTGTACTACGGA-3'	135	PCR
(exon 3)	Reverse	5'-CTGGATTTTGAGCCTGAATT-3'		
ARMC12	Forward	5'-GAACACTCCATCAAAGTACT-3'	174	PCR
(exon 4)	Reverse	5'-CTGTGCCAGGATGTAGTCTG-3'		
ARMC12	Forward	5'-GTGCAAGCCGTACGACTGCT-3'	72	PCR
(exon 5)	Reverse	5'-CTGGCAGTTGAGAATGTCAT-3'		
ARMC12	Forward	5'-GTTCACTCCAACTTCCTAAA-3'	376	PCR
(exon 6)	Reverse	5'-TTCAAGTTTCTCTCCTATAC-3'		
RBBP4	Forward	5'-CAGCATTCATCGACTTGTCCT-3'	108	qPCR
	Reverse	5'-TGTGACGCATCAAACTGAGCA-3'		
EZH2	Forward	5'-AATCAGAGTACATGCGACTGAGA-3'	141	qPCR
	Reverse	5'-GCTGTATCCTTCGCTGTTTCC-3'		
SUZ12	Forward	5'-TTGCAGCTTACGTTTACTGGTT-3'	152	qPCR
	Reverse	5'-GGAACTTGCCTTATTGGACAACT-3'		
CADM1	Forward	5'-GCCCAATTCCGCAGAAGAAC-3'	134	qPCR
	Reverse	5'-AGCACAGCATGGCGAACACC-3'		
EGLN3	Forward	5'-ATCAGCTTCCTCCTGTCCCT-3'	126	qPCR
	Reverse	5'-ATAACCTGTTCCATTTCCCG-3'		
HRK	Forward	5'-TGCACCAGCGCACCATGT-3'	142	qPCR
	Reverse	5'-AAGTTCCGCCTGCCGAGC-3'		
HS6ST3	Forward	5'-CCGCAACCACAGCCACAC-3'	190	qPCR
	Reverse	5'-CCCAGACCAGTCATCCCC-3'		
SMAD9	Forward	5'-GCTGGAGTGCTGTGAGTTC-3'	207	qPCR
	Reverse	5'-GGATAGGTGGCGTTGTGTG-3'		
GAPDH	Forward	5'-AGAAGGCTGGGGCTCATTTG-3'	258	qPCR
	Reverse	5'-AGGGGCCATCCACAGTCTTC-3'		
CADM1	Forward	5'-ACAAAACAATCTGGGAGAAGTG-3'	204	ChIP
	Reverse	5'-GACAAGCGGAGGAGCCTGAGCA-3'		
EGLN3	Forward	5'-GGTGTCTTTCCTTATTTACCCC-3'	152	ChIP
	Reverse	5'-CTCCTCAAGCACATAGTGCCTT-3'		
HRK	Forward	5'-TTTCCAATCCGACAGCCACTC-3'	121	ChIP
	Reverse	5'-CAGGTTTCATTCTCCCCCACA-3'		
HS6ST3	Forward	5'-GGGCTGGACTGTCTAGTGCTGA-3'	160	ChIP
	Reverse	5'-TGGTGTTCGAGTTTTATGGTGG-3'		
SMAD9	Forward	5'-ACCCCACCTCTGAGATTTCTTG-3'	226	ChIP
	Reverse	5'-CAGTTGCTCTGTCACTGTCCAT-3'		

ARMC12, armadillo repeat containing 12; RBBP4, retinoblastoma binding protein 4; EZH2, enhancer of zeste 2 polycomb repressive complex 2 subunit; SUZ12, suppressor of zeste 12 homolog; CADM1, cell adhesion molecule 1; EGLN3, egl-9 family hypoxia inducible factor 3; HRK, harakiri, BCL2 interacting protein; HS6ST3, heparan sulfate 6-O-sulfotransferase 3; SMAD9, SMAD family member 9; GAPDH, glyceraldehyde 3-phosphate dehydrogenase; ChIP, chromatin immunoprecipitation.
